# Estimating the Minimal Number of Repeated Examinations for Random Responsiveness With the Coma Recovery Scale—Revised as an Example

**DOI:** 10.3389/fnint.2021.685627

**Published:** 2021-07-08

**Authors:** Hao Yang, Chengyin Ye, Xiaochen Liu, Lingxiu Sun, Anqi Wang, Jing Wang, Nantu Hu, Xiaohua Hu, Olivia Gosseries, Steven Laureys, Haibo Di, Jiqian Fang

**Affiliations:** ^1^International Unresponsive Wakefulness Syndrome and Consciousness Science Institute, Hangzhou Normal University, Hangzhou, China; ^2^Department of Health Management, School of Public Health, Hangzhou Normal University, Hangzhou, China; ^3^Hangzhou Wujing Hospital, Hangzhou, China; ^4^Coma Science Group, GIGA-Consciousness and Centre du Cerveau, University and University Hospital of Liège, Liège, Belgium; ^5^School of Public Health, Sun Yat-sen University, Guangzhou, China

**Keywords:** repeated examinations, random responsiveness, diagnosis, Coma Recovery Scale-Revised, disorders of consciousness, minimally conscious state

## Abstract

**Objective:** The aim of this study was to develop a general method to estimate the minimal number of repeated examinations needed to detect patients with random responsiveness, given a limited rate of missed diagnosis.

**Methods:** Basic statistical theory was applied to develop the method. As an application, 100 patients with disorders of consciousness (DOC) were assessed with the Coma Recovery Scale–Revised (CRS-R). DOC patients were supposed to be examined for 13 times over 20 days, while anyone who was diagnosed as a minimally conscious state (MCS) in a round would no longer be examined in the subsequent rounds. To test the validation of this method, a series of the stochastic simulation was completed by computer software under all the conditions of possible combinations of three kinds of distributions for *p*, five values of *p*, and four sizes of the sample and repeated for 100 times.

**Results:** A series of formula was developed to estimate the probability of a positive response to a single examination given by a patient and the minimal number of successive examinations needed based on the numbers of patients detected in the first *i* (*i* =1, 2,.) rounds of repeated examinations. As applied to the DOC patients assessed with the CRS-R, with a rate of missed diagnosis < 0.0001, the estimate of the minimal number of examinations was six in traumatic brain injury patients and five in non-traumatic brain injury patients. The outcome of the simulation showed that this method performed well under various conditions possibly occurring in practice.

**Interpretation:** The method developed in this paper holds in theory and works well in application and stochastic simulation. It could be applied to any other kind of examinations for random responsiveness, not limited to CRS-R for detecting MCS; this should be validated in further research.

## Introduction

In clinical practice, bedside evaluation of patients has always been regarded as the “gold standard” (Giacino et al., 2009). However, the fluctuation of behavioral responses of patients is very common, especially in the minimally conscious state (MCS) (Giacino et al., 2002). Patients with MCS are defined as those who exhibit inconsistent but repeatable purposeful behaviors (Giacino et al., 2002). That is to say, the fluctuations in the responsiveness of MCS patients are inherent (Majerus et al., 2005), and this is due to the patient's motor or language impairments and vigilance fluctuations (Seel et al., 2010). Both unresponsive wakefulness syndrome/vegetative state (UWS/VS) (Laureys et al., 2010) and MCS are referred to as “disorders of consciousness” (DOC), but clinicians often make mistakes when differentiating between these patients. Many studies have shown that the fluctuations of MCS often leads to missed diagnosis (the missed diagnosis rate may be higher than 35%) (Stender et al., 2014, van Erp et al., 2015), and that such diagnostic errors can affect patient outcomes and treatment decisions (Demertzi et al., 2011). Especially when DOC patients were tested only once, even when using the most sensitive scale of the Coma Recovery Scale-Revised (CRS-R) (Giacino et al., 2004), a single assessment resulted in a 25% missed diagnosis (Wang et al., 2020). Therefore, recent guidelines have emphasized the importance of repeated assessments to increase the accuracy of clinical diagnosis (Giacino et al., 2018; Kondziella et al., 2020).

Wannez et al. assessed 123 chronic DOC patients using the CRS-R at least six times over a 10-day period, and their results suggest to perform at least five assessments in each patient with DOC within a short time interval. But this conclusion was statistically analyzed based on the best diagnosis of the six tests (Wannez et al., 2017). There was still a 10% missed diagnosis at the fifth assessment when compared with the best diagnosis from the seven tests in the subgroup (Wannez et al., 2017). So, it is questionable whether five assessments are sufficient. In fact, the number of repeated examinations needed depends on the probability of MCS patients giving a positive response at a single examination.

The aim of this study is to develop a general method to estimate the minimal number of repeated examinations needed to detect patients with random responsiveness, given a limited rate of missed diagnosis and to validate the method by computer stochastic simulation.

To avoid abstract mathematical derivation, we will use the language of detecting MCS from DOC to develop and explain the method. Considering that there are two types of DOC patients (including MCS and UWS/VS), we assume that there are *n* independent patients with MCS and *N*−*n* independent patients with UWS/VS in a group of *N* patients with DOC; and the probability of an MCS patient giving a positive response to a single CRS-R assessment is *p* in theory. We will develop a series of statistical formulas for the estimation of *n* and *p*, and, hence, the minimal number of successive examinations needed *k*_min_ with a rate of missed diagnosis less than a given α, say α = 0.0001.

## Materials and Methods

### Bedside Examinations

#### The DOC Patients

They were part of the patients treated in the rehabilitation department of Hangzhou Wujing Hospital. The inclusion criteria for this project were as follows: (1) over 18 years old, (2) injured for at least 28 days, and (3) no drugs affecting conscious expression (e.g., no neuromuscular blockers or sedatives within 72 h of enrollment). Comatose patients and patients who emerged from MCS were excluded.

#### Procedure of Examination

The standard operating procedure of the CRS-R is the same as the study of Wannez et al. (2017). The difference is the arrangement of appraisers and the frequency of appraisals. Examinations were performed independently by two appraisers who were well-trained and experienced in the use of the CRS-R, and recorded by a video. The results given by the two appraisers were compared right after each round; if they were not consistent, the final diagnosis was determined by discussion among the two appraisers and the third person incorporating with the recorded video. If the patient was first diagnosed as MCS in a round, then he or she would no longer be examined in the subsequent rounds; otherwise, the behavioral examination with the CRS-R was continued until the 13th round for a maximum of 20 days.

This study protocol was approved by the Ethics Committee of Hangzhou Normal University. Written informed consent was signed by the legal guardians of the patients enrolled in this study.

### Development of Statistical Formulas

#### The Assumptions

The assumptions of the formulas to be developed are the following: (1) The number of MCS patients *n* is big enough. (2) Each of the MCS patients may independently give a positive response to a single examination with the same constant probability *p* at any time; and each of non-MCS patients may never give any positive response to the examination. (3) The examination is administered independently for several rounds according to unique standard criteria; if someone successively gives a negative response up to the (*i* – 1)-th round of examination but gives a positive response at the *i*–th round, then the examination for this person is ended; otherwise, he or she keeps going. (4) The examination for the whole group is supposed to stop at the *k*_min_-th round with a small rate of missed diagnosis controlled by a given α . The key target is to determine the size of *k*_min_ based on the observed numbers of new cases, giving positive response in the successive rounds, *a*_1_, *a*_2_, ⋯ , *a*_*k*_min__, and the bi-product is the estimate of the probability *p*.

#### Data and Formulas

In Table 1, the second and third columns show the number of new cases of MCS, giving positive responses and the total number of MCS patients really assessed in each round of examinations. If the total number of MCS patients is big enough, based on the data of each round, any proportion in the fourth column can be an estimate of the probability of positive response to a single examination given by an MCS patient; and, in theory, based on the data of a series of examinations from the first up to the *i*-th round, the pooled estimate in the fifth column is better than the estimate based on the data of the *i*-th round only, *i* = 1, 2…

**Table 1:** Pooled estimate for the probability of positive response to a single examination given by an MCS patient in theory.

**No. of rounds *i***	**No. of MCSs giving positive response**	**Total no of MCS assessed**.	**Proportion**	**Pooled estimate for the probability of positive response to a single examination**
				**given by an MCS**
1	*a*_1_	*n*	$\frac{a_{1}}{n}$	$\frac{a_{1}}{n}$
2	*a*_2_	*n* − *a*_1_	$\frac{a_{2}}{n-a_{1}}$	$\frac{a_{1}+a_{2}}{n+\left(n-a_{1}\right)}$
3	*a*_3_	*n* − *a*_1_ − *a*_2_	$\frac{a_{3}}{n-a_{1}-a_{2}}$	$\frac{a_{1}+a_{2}+a_{3}}{n+\left(n-a_{1}\right)+\left(n-a_{1}-a_{2}\right)}$
…	…	…	…	…
*i*	*a*_*i*_	*n* − *a*_1_ − … − *a*_*i*−1_	$\frac{a_{i}}{n-a_{1}-\cdots -a_{i-1}}$	$\frac{a_{1}+a_{2}+\cdots +a_{i}}{n+\left(n-a_{1}\right)+\cdots +\left(n-a_{1}-a_{2}-\cdots -a_{i-1}\right)}$

*MCS, minimally conscious state*.

Since both of *a*_1_/*n* and any of the formulas in the 5-th column of Table 1 approximate to the same probability of positive response to a single examination given by a MCS patient, we have


$$\begin{array}{l} \frac{a_{1}}{n}\approx \frac{a_{1}+a_{2}}{n+\left(n-a_{1}\right)},\\ \frac{a_{1}}{n}\approx \frac{a_{1}+a_{2}+a_{3}}{n+\left(n-a_{1}\right)+\left(n-a_{1}-a_{2}\right)} \end{array}$$

and


$$\begin{array}{l} \frac{a_{1}}{n}\approx \frac{a_{1}+\cdots +a_{i}}{n+\left(n-a_{1}\right)+\cdots \left(n-a_{1}-\cdots -a_{i-1}\right)} \end{array}$$

Denote their solutions of *n*, respectively, by


$$\begin{array}{l} {\hat{n}}_{2}\approx \frac{a_{1}^{2}}{a_{1}-a_{2}},\;{\hat{n}}_{3}\approx \frac{a_{1}\left(2a_{1}+a_{2}\right)}{2a_{1}-a_{2}-a_{3}} \end{array}$$

and


$$\begin{array}{l} {\hat{n}}_{i}\approx \frac{a_{1}\left[\left(i-1\right)a_{1}+\left(i-2\right)a_{2}+\cdots +a_{i-1}\right]}{\left(i-1\right)a_{1}-a_{2}-\cdots -a_{i}},\;{\hat{p}}_{i}=\frac{a_{1}}{{\hat{n}}_{i}} \end{array}$$

These formulas have been summarized in Table 2.

**Table 2:** Estimation for a total number of MCS patients and their probability of giving positive response to a single examination.

**No. of rounds**	**No. of MCSs giving positive response**	**Estimated total no. of MCSs**	**Estimated probability of positive response to a single examination given by an MCS**
***i***	***a*_*i*_**	${\hat{n}}_{i}$	${\hat{p}}_{i}$
1	*a*_1_		
2	*a*_2_	${\hat{n}}_{2}=\frac{a_{1}^{2}}{a_{1}-a_{2}}$	${\hat{p}}_{2}=\frac{a_{1}-a_{2}}{a_{1}}$
3	*a*_3_	${\hat{n}}_{3}=\frac{a_{1}\left(2a_{1}+a_{2}\right)}{2a_{1}-a_{2}-a_{3}}$	${\hat{p}}_{3}=\frac{2a_{1}-a_{2}-a_{3}}{2a_{1}+a_{2}}$
…	…	…	…
*i*	*a*_*i*_	${\hat{n}}_{i}=\frac{a_{1}\left[\left(i-1\right)a_{1}+\left(i-2\right)a_{2}+\cdots +a_{i-1}\right]}{\left(i-1\right)a_{1}-a_{2}-\cdots -a_{i}}$	${\hat{p}}_{i}=\frac{\left(i-1\right)a_{1}-a_{2}-\cdots -a_{i}}{\left(i-1\right)a_{1}+\left(i-2\right)a_{2}+\cdots +a_{i-1}}$

*MCS, minimally conscious state*.

The possible minimal number of rounds *k*_min_ can be estimated right after any *i*-th round by finding a minimal ${\hat{k}}_{i,\min}$ such that


$$\begin{array}{l} {\left(1-{\hat{p}}_{i}\right)}^{k}\geq \alpha ,\;k=i,i+1,\cdots {\hat{k}}_{i,\min}-1;\;{\left(1-{\hat{p}}_{i}\right)}^{{\hat{k}}_{i,\min}}<\alpha . \end{array}$$

When ${\hat{k}}_{i,\min}=i$, we have ${\hat{k}}_{\min}=i$ finally.

### Validation by Stochastic Simulation

#### The “DOC Patients”

Set up *N* binary variables *Y*_*j*_, *j* = 1, 2, ⋯ , *n, n*+1, ⋯*N* representing *N* “DOC patients,” *Y*_*j*_ = 1, *j* = 1, 2, ⋯ , *n* representing *n* “MCS patients,” and *Y*_*j*_≡0, *j* = *n*+1, ⋯*N* representing *N*-*n* “non-MCS patients.”

#### The Inherent Ability on Response

The probabilities of *j*-th patient giving a positive response to a single examination *p*_*j*_, *j* = 1, 2, ⋯ , *n* representing his or her inherent ability on response:

1. Constant *p*_*j*_≡*p* to show that this method works well for a large sample (say *n* = 500, 1,000) and applicable to a small sample (say *n* = 100 or 50);
2. *p*_*j*_ following a normal distribution *N*(*p*, σ^2^), to show that this method still works well when there is some normality-like variation among patients (say σ = 0.1, 0.2, 0.3); the values of *p*_*j*_, *j* = 1, 2, ⋯ , *n* in each time of simulation were generated by statistical software;
3. *p*_*j*_ following a uniform distribution *U*(*p*−δ, *p*+δ), to show that this method still works well when there is uniform-like variation among patients (say δ = 0.1, 0.2, 0.3); the values of *p*_*j*_, *j* = 1, 2, ⋯ , *n* in each time of simulation were generated by statistical software.

For non-MCS patients, let *p*_*j*_≡0, *j* = *n*+1, ⋯*N* to ensure that they will never give any positive response to the “examination.”

####  “Examination” and “Responses”

During *i*-th round of “examination,” generate *a* random number *r*_*ij*_ in the interval of [0, 1] for the *j*-th patient; if *p*_*j*_≥*r*_*ij*_, let Ŷ_*ij*_ = 1, indicating a “positive response” given by the *j*-th patient in the *i*-th round; otherwise, Ŷ_*ij*_ = 0.

After *i*-th round of “examination,” define $a_{i}=\underset{j}{\sum }{Ŷ}_{ij}$ as the total number of MCS patients diagnosed in this round.

From each time of simulation, we can obtain an estimated probability $\hat{p}$, the minimal number of examinations ${\hat{k}}_{\text{min}}$, the total numbers of diagnosis, $\hat{n}=\overset{{\hat{k}}_{\min}}{\underset{i=1}{\sum }}a_{i}$, the rate of missed diagnosis $\left(n-\hat{n}\right)/n$.

#### Repeated Simulation and the Rate of Missed Diagnosis

In order to see the robustness of this method, we have repeated the simulation for 100 times and calculated the means of $\hat{p}$, ${\hat{k}}_{\text{min}}$, $\left(n-\hat{n}\right)/n$ and corresponding 95%-confidence intervals.

## Results

To easily understand the procedure of this method, we would like to show the results on real data first, and then those on the simulation.

### Outcome of Bedside Examinations

A total of 100 DOC patients, including 50 traumatic brain injury (TBI) patients and 50 non-traumatic brain injury (NTBI) patients were recruited and assessed with the CRS-R. The TBI patients consisted of 40 males and 10 females aged 18–77 years (standard deviation = 13.0); the time after the injury onset was from 1 to 24 months (SD = 4.4). The NTBI patients consisted of 34 males and 16 females aged 26–83 years (standard deviation = 14.9); the time after the injury onset was from 1 to 27.8 months (standard deviation = 4.8). There was no significant difference in age (the detailed information on patients is shown in Supplementary Material A). The above-developed method had been applied to the observed data with **α** = 0.0001 being assigned. Table 3 shows the diagnostic results of all the DOC patients after each examination.

**Table 3:** The data collected from the 13 rounds of successive examinations.

**Group**	**No. of MCSs giving positive response in each round of examinations**													**MCS**	**UWS/VS**	**Total**
	***a*_1_**	***a*_2_**	***a*_3_**	***a*_4_**	***a*_5_**	***a*_6_**	***a*_7_**	***a*_8_**	***a*_9_**	***a*_10_**	***a*_11_**	***a*_12_**	***a*_13_**			
TBI	30	3	3	2	0	0	0	0	0	0	0	0	0	38	12	50
NTBI	29	3	2	1	0	0	0	0	0	0	0	0	0	35	15	50

*MCS, minimally conscious state; UWS/VS, unresponsive wakefulness syndrome/vegetative state; TBI, traumatic brain injury; NTBI, non-traumatic brain injury*.

#### For TBI Patients

After completing the first 2 rounds of examinations we obtained the numbers of MCSs giving positive response *a*_1_ = 30 and *a*_2_ = 3, using the formulas in the second row of Table 2, we had the estimated *n* and *p* as


$$\begin{array}{l} {\hat{n}}_{2}\approx \frac{a_{1}^{2}}{a_{1}-a_{2}}=\frac{900}{27}=33.33,\\ {\hat{p}}_{2}\approx \frac{a_{1}}{{\hat{n}}_{2}}=\frac{30}{33.33}=0.9001 \end{array}$$

Since


$$\begin{array}{l} {\left(1-{\hat{p}}_{2}\right)}^{k}\geq 0.0001,k=2,3,4,\\ {\left(1-{\hat{p}}_{2}\right)}^{5}=0.00001<0.0001 \end{array}$$

the examination should be kept on going, and might be ended at the 5-th round; and the total number of MCS patients in this group of DOCs might be around 34.

After completing the 3rd round, we obtained *a*_3_ = 3, and


$$\begin{array}{l} {\hat{n}}_{3}\approx \frac{a_{1}\left(2a_{1}+a_{2}\right)}{2a_{1}-a_{2}-a_{3}}=\frac{30\times 63}{54}=35,\\ {\hat{p}}_{3}\approx \frac{a_{1}}{{\hat{n}}_{3}}=\frac{30}{35}=0.8571 \end{array}$$

Since


$$\begin{array}{l} {\left(1-{\hat{p}}_{3}\right)}^{i}\geq 0.0001,\;i=3,4,\\ {\left(1-{\hat{p}}_{3}\right)}^{5}=0.00006<0.0001 \end{array}$$

the examination should be kept on going, and might be ended at the 5-th round; and the total number of MCS patients in this group of DOCs might be around 35.

After completing the 4-th round, we obtained *a*_4_ = 2, and


$$\begin{array}{l} {\hat{n}}_{4}\approx \frac{a_{1}\left(3a_{1}+2a_{2}+a_{3}\right)}{3a_{1}-a_{2}-a_{3}-a_{4}}=\frac{30\times 99}{82}=36.22,\\ {\hat{p}}_{4}\approx \frac{a_{1}}{{\hat{n}}_{4}}=\frac{30}{36.22}=0.8283 \end{array}$$

Since


$$\begin{array}{l} {\left(1-{\hat{p}}_{4}\right)}^{i}\geq 0.0001,i=4,5,\\ {\left(1-{\hat{p}}_{4}\right)}^{6}=0.00003<0.0001 \end{array}$$

the examination should be kept on going, and might be ended at the 6-th round, and the total number of MCS patients in this group of DOCs might be around 37.

After completing the 5-th round, we obtained *a*_5_ = 0, and


$$\begin{array}{l} {\hat{n}}_{5}\approx \frac{a_{1}\left(4a_{1}+3a_{2}+2a_{3}+a_{4}\right)}{4a_{1}-a_{2}-a_{3}-a_{4}-a_{5}}=\frac{30\times 137}{112}=36.70,\\ {\hat{p}}_{5}\approx \frac{a_{1}}{{\hat{n}}_{5}}=\frac{30}{36.70}=\;0.8175 \end{array}$$

Since


$$\begin{array}{l} {\left(1-{\hat{p}}_{5}\right)}^{5}=0.00020,\\ {\left(1-{\hat{p}}_{5}\right)}^{6}=0.00004<0.0001 \end{array}$$

the examination should be kept on going, and perhaps ended at the 6-th round; and the total number of MCS patients in this group of DOCs might be around 37.

After completing the 6-th round, we obtained *a*_6_ = 0, and


$$\begin{array}{l} {\hat{n}}_{6}\approx \frac{a_{1}\left(5a_{1}+4a_{2}+3a_{3}+2a_{4}{+a}_{5}\right)}{5a_{1}-a_{2}-a_{3}-a_{4}-a_{5}-a_{6}}=\frac{30\times 175}{142}=36.97,\\ {\hat{p}}_{6}\approx \frac{a_{1}}{{\hat{n}}_{6}}=\frac{30}{36.97}=\;0.8114 \end{array}$$

Since


$$\begin{array}{l} {\left(1-{\hat{p}}_{6}\right)}^{6}=0.00005<\;0.0001, \end{array}$$

the examination could be ended at this round ${\hat{k}}_{\text{min}}=6$.

And up to this round, the total number of MCS patients had been detected in the group of DOCs was


$$\begin{array}{l} \hat{n}=a_{1}+a_{2}+a_{3}+a_{4}+a_{5}+a_{6}=38 \end{array}$$

Following the guidance of our developed method, based on six rounds of examination, we could conclude with a missed diagnosis rate <0.01% that there were 38 patients with MCS in total out of *N* = 50 DOCs in this group of patients with TBI and the probability of the patients with MCS giving positive response in a single examination was about 81%. This conclusion was supported by the real data collected from 13 rounds of successive examinations (Table 3), where the number of detected MCSs was *a*_1_ = 30, *a*_2_ = 3, *a*_3_ = 3, *a*_4_ = 2, *a*_5_ =…= *a*_13_ = 0, respectively.

#### For NTBI Patients

After completing the first two rounds of examination we obtained the numbers of MCSs giving positive response *a*_1_ = 29 and *a*_2_ = 3, using the formulas in the second row of Table 2, we had


$$\begin{array}{l} {\hat{n}}_{2}=\frac{a_{1}^{2}}{a_{1}-a_{2}}=\frac{29^{2}}{26}=32.35,\\ {\hat{p}}_{2}\approx \frac{a_{1}}{{\hat{n}}_{2}}=\frac{29}{32.35}=0.8966 \end{array}$$

Since


$$\begin{array}{l} {\left(1-{\hat{p}}_{2}\right)}^{i}\geq 0.0001,\;i=2,3,4,\\ {\left(1-{\hat{p}}_{2}\right)}^{5}=0.000012<\;0.0001 \end{array}$$

the examination should be kept on going, and might be ended at the 5-th round; and the total number of MCS patients in this group of DOCs might be 33.

After completing the 3rd round, we obtained *a*_3_ = 2, and


$$\begin{array}{l} {\hat{n}}_{3}=\frac{a_{1}\left(2a_{1}+a_{2}\right)}{2a_{1}-a_{2}-a_{3}}=\frac{29\times 61}{53}=33.38,\\ {\hat{p}}_{3}\approx \frac{a_{1}}{{\hat{n}}_{3}}=\frac{29}{33.38}=\;0.8689 \end{array}$$

Since


$$\begin{array}{l} {\left(1-{\hat{p}}_{3}\right)}^{i}\geq 0.0001,\;i=3,4,\\ {\left(1-{\hat{p}}_{3}\right)}^{5}=0.000039<\;0.0001 \end{array}$$

the examination should be kept on going, and might be ended at the 5-th round; and the total number of MCS patients in this group of DOCs might be 34.

After completing the 4-th round, we obtained *a*_4_ = 1, and


$$\begin{array}{l} {\hat{n}}_{4}=\frac{a_{1}\left(3a_{1}+2a_{2}+a_{3}\right)}{3a_{1}-a_{2}-a_{3}-a_{4}}=\frac{29\times 95}{81}=34.01,\\ {\hat{p}}_{4}\approx \frac{a_{1}}{{\hat{n}}_{4}}=\frac{29}{34.01}=0.8526 \end{array}$$

Since


$$\begin{array}{l} {\left(1-{\hat{p}}_{4}\right)}^{4}=0.000472\geq 0.0001,\\ {\left(1-{\hat{p}}_{4}\right)}^{5}=0.000070<\;0.0001 \end{array}$$

the examination could be ended at 5-th round; and the total number of MCS patients in this group of DOCs might be 35.

After completing the 5-th round, we obtained *a*_5_ = 0, and


$$\begin{array}{l} {\hat{n}}_{5}=\frac{a_{1}\left(4a_{1}+3a_{2}+2a_{3}+a_{4}\right)}{4a_{1}-a_{2}-a_{3}-a_{4}-a_{5}}=\frac{29\times 130}{110}=34.27,\\ {\hat{p}}_{5}\approx \frac{a_{1}}{{\hat{n}}_{5}}=\frac{29}{34.27}=0.8462 \end{array}$$

Since


$$\begin{array}{l} {\left(1-{\hat{p}}_{5}\right)}^{5}=0.000086<0.0001 \end{array}$$

the examination could be ended at this round ${\hat{k}}_{\text{min}}=5$ and up to this round, the total number of MCS patients had been detected in the group of DOCs was


$$\begin{array}{l} \hat{n}=a_{1}+a_{2}+a_{3}+a_{4}+a_{5}=35 \end{array}$$

Following the guidance of our developed method, based on the five rounds of examination, we could conclude with a missed diagnosis rate <0.01% that, out of *N* = 50 DOCs in this group of patients with non-TBI, there were 35 patients with MCS in total; and the probability of the patients with MCS giving positive response in a single examination was about 85%. This conclusion was also supported by the real data collected from the 13 rounds of successive examinations (Table 3), where the number of detected MCSs ws *a*_1_ = 29, *a*_2_ = 3, *a*_3_ = 2, *a*_4_ = 1, *a*_5_ =…= *a*_13_ = 0, respectively.

### Outcomes of Simulation

The process of a single time of simulation included three conditions of probability *p* mentioned above; under each condition, *p* = 0.5, 0.6, 0.7, 0.8, 0.9 were simulated with sample size *n* = 50, 100, 500, 1, 000 respectively. The detailed outcome of the simulation has been packaged in a file of Supplementary Material B, which will be available upon request. The key findings were summarized in Tables 4–**6**.

**Table 4:** Estimated probabilities $\hat{p}$ for *p*_*i*_≡*p*, $p_{i}\sim N\left(p,0.3^{2}\right)$ and *p*_*i*_~*U*(*p*−0.3, *p*+0.3)^*^.

**p**		**0.5**			**0.6**			**0.7**			**0.8**			**0.9**		
**Dist**.		***C***	***N***	***U***	***C***	***N***	***U***	***C***	***N***	***U***	***C***	***N***	***U***	***C***	***N***	***U***
*n*	1,000	0.497	0.501	0.499	0.599	0.600	0.601	0.699	0.701	0.700	0.799	0.800	0.800	0.899	0.899	0.900
	500	0.498	0.501	0.499	0.600	0.599	0.600	0.701	0.703	0.702	0.800	0.798	0.799	0.898	0.901	0.901
	100	0.507	0.499	0.506	0.610	0.596	0.601	0.711	0.703	0.702	0.807	0.800	0.800	0.900	0.893	0.898
	50	0.515	0.509	0.500	0.601	0.605	0.611	0.707	0.702	0.700	0.807	0.801	0.805	0.901	0.901	0.900

* *The distributions p_i_≡p, $p_{i}\sim N\left(p,0.3^{2}\right)$ and p_i_~U(p−0.3, p+0.3) are expressed by C, N, and U, respectively, for short*.

#### Estimation on p, *k*_min_ and Missed Diagnosis Rate

Table 4 gives the estimated probabilities $\hat{p}$. One can see that the maximal error $\left|p-\hat{p}\right|$ is 0.003 for a bigger sample size (500, 1,000), and 0.015 for a smaller sample size (50, 100), which are not significant in practice.

Table 5 gives the estimated minimal number of repeated examinations needed ${\hat{k}}_{\min}$. One can see that the maximal error $k_{\text{min}}-{\hat{k}}_{\text{min}}$ is 0.5 for a bigger sample size (500, 1,000) and 0.7 for a smaller sample size (50, 100), which reminds us that ${\hat{k}}_{\min}$is a conservative estimate, and using ${\hat{k}}_{\min}$ as the estimate might be more safe in practice.

**Table 5:** The numbers of repeated examination ${\hat{k}}_{min}$ for *p*_*i*_≡*p*,*p*~*N*(*p*, 0.3^2^) and *p*_*i*_~*U*(*p*−0.3,*p*+0.3)^*^.

**p**		**0.5**			**0.6**			**0.7**			**0.8**			**0.9**		
**Dist**.		***C***	***N***	***U***	***C***	***N***	***U***	***C***	***N***	***U***	***C***	***N***	***U***	***C***	***N***	***U***
*n*	1,000	13.7	13.9	13.8	10.6	10.6	10.5	8.1	8.1	8.1	6.1	6.1	6.1	4.6	4.5	4.5
	500	14.0	13.8	13.9	10.6	10.6	10.5	8.1	8.1	8.1	6.2	6.2	6.2	4.5	4.5	4.5
	100	13.8	14.3	13.7	10.5	10.9	10.7	8.0	8.1	8.2	6.1	6.4	6.3	4.5	4.6	4.5
	50	13.3	13.4	14.3	10.8	10.5	10.4	8.1	8.3	8.3	6.2	6.2	6.1	4.5	4.5	4.5
*k*_min_		14			11			8			6			5		

* *The distributions *p*_*i*_≡*p*, $p_{i}\sim N\left(p,0.3^{2}\right)$ and *p*_*i*_~*U*(*p*−0.3, *p*+0.3) are expressed by *C, N*, and *U* for short in this table*.

Table 6 gives the average rate of missed diagnosis. One can see that, since we controlled the rate of missed diagnosis by α = 0.0001, the real rates of missed diagnosis were mostly <0.0001 for bigger sample (500, 1,000), but, occasionally, it might be 0.0002 for a bigger sample and even 0.00640 for *p* = 0.5 and *n* = 50.

**Table 6:** The average rate of missed diagnosis for *p*_*i*_≡*p*, $p_{i}\sim N\left(p,0.3^{2}\right)$ and *p*_*i*_~*U*(*p*−0.3, *p*+0.3)^*^.

**p**		**0.5**			**0.6**			**0.7**			**0.8**			**0.9**		
**Dist**.		***C***	***N***	***U***	***C***	***N***	***U***	***C***	***N***	***U***	***C***	***N***	***U***	***C***	***N***	***U***
*n*	1,000	0.00010	0.00013	0.00013	0.00006	0.00010	0.00006	0.00008	0.00008	0.00005	0.00009	0.00005	0.00005	0.00009	0.00016	0.00008
	500	0	0.00010	0.00004	0.00008	0.00008	0.00010	0.00008	0.00010	0.00006	0.00006	0.00014	0.00006	0	0.00020	0.00006
	100	0.00010	0.00030	0.00030	0	0.00020	0	0.00010	0.00010	0	0.00030	0.00010	0.00020	0	0	0
	50	0.00600	0.00640	0.00020	0.00020	0.00160	0.00160	0	0.00060	0.00020	0.00060	0.00060	0.00040	0	0	0.00060

* *The distributions p_i_≡p, $p_{i}\sim N\left(p,0.3^{2}\right)$ and p_i_~U(p−0.3, p+0.3) are expressed by C, N, and U, respectively, for short*.

#### The Robustness of the Estimation

In Tables 4–6, through the comparison among *p*_*i*_≡*p*,$p_{i}\tilde{\;}N\left(p,0.3^{2}\right)$ and $p_{i}\tilde{\;}U\left(p-0.3,p+0.3\right)$ we can see that the estimated *p*, *k*_min_ and the missed diagnosis rate are fairly stable; the absolute errors $\left|p-\hat{p}\right|$ and $k_{\text{min}}-{\hat{k}}_{\text{min}}$ are not affected by whether *p*_*j*_ being constant or varied as we simulated and are not affected by whether *p*_*j*_ being normality-like varied or uniform-like varied as we simulated.

## Discussion

There is a consensus that the fluctuations in responsiveness are inherent to MCS patients (Giacino et al., 2002, 2009; Majerus et al., 2005), so people should repeat the examination to obtain a reliable diagnosis while using the CRS-R. We assume that there is a probability *p* for a patient with MCS, giving a positive response to a single examination. In theory, the minimal number *k*_min_ of repeated examinations needed for a homogeneous MCS population depends on the size of *p*; if *p* is bigger, *k*_min_ is less, and, otherwise, *k*_min_ should be bigger. We have developed a series of statistical formulas to estimate the probability *p*, the total number *n* of MCSs, and the necessary minimal number of *k*_min_ of repeated examinations based on the numbers of new cases detected in successive rounds of examinations for a group of patients in DOC. These formulas had been applied to our clinical practice along with the following procedure: After completing the *i*-th round of examinations (*I* = 2, 3…), based on the numbers of new cases of MCS detected up to the *i*-th round (*a*_1_, *a*_2_,…, *a*_*i*_), to calculate ${\hat{n}}_{i}$ and ${\hat{p}}_{i}$ according to the formulas in the *i*-th row of Table 1 and given a very small probability α, if ${\left(1-{\hat{p}}_{i}\right)}^{i}\geq \alpha$, then the examination should be continued; otherwise, ended. Meanwhile, to estimate the minimal number needed for repeated examinations at the *i*-th round by finding a minimal *k*_*i*, min_ such that $\;{\left(1-{\hat{p}}_{i}\right)}^{{\hat{k}}_{i,\min}}<\alpha$ , as a result given, α= 0.0001, 38 MCSs out of a group of 50 TBI patients were detected through the first six rounds of successive examinations without any more patients giving positive response in the following seven rounds, while 35 MCSs out of a group of 50 NTBI patients were detected in the first five rounds without any more positive response in the following eight rounds.

Based on our data, in case the patient's etiology was NTBI, the probability for a patient with MCS giving a positive response was about 85%, and the corresponding number of repeated rounds needed was 5. This number coincides with the number suggested in the article of Wannez et al. (2017). However, for TBI patients, the probability of the patients with MCS giving positive response in a single examination was about 81%, and the corresponding number of repeated rounds needed was 6. This coproduct might be important. As we all know, patients with different kinds of etiology should be managed in different ways. For example, the ethical and legal issues concerning treatment withdrawal can be discussed when the NTBI patients were injured after 3 months without recovery, while the TBI patients were injured after 12 months without recovery (Sazbon and Groswasser, 1991). This result also reminds us that the patients with TBI have a higher degree of behavioral instability, suggesting that we should carefully monitor the behavioral performance of such patients in clinical practice.

More than the application for the real data of bedside examination, this method had been validated by a series of stochastic simulations. Each simulation explored all the conditions of the combination of three kinds of distributions for *p*, five values of *p*, and four sizes of the sample and repeated for 100 times. The outcome of the simulation was fairly enlightening; the estimation on *p* was satisfied with very small absolute errors even when the sample size is small as *n* = 50; the estimation on*k*_min_ was fine for a bigger sample, and using *k*_min_+1 as the estimate might be safer for the small sample. Although we controlled for the rate of missed diagnosis by α = 0.0001, the real rate of missed diagnosis was not necessarily <0.0001, occasionally might be 0.0002 and even 0.00640 for *p* = 0.5 and *n* = 50. Therefore, in practice, one can still assign the limit as α = 0.0001 but should not clearly claim the rate of missed diagnosis being <0.0001, especially for a small sample size as *n* = 50. For safety, one may assign α = 0.00001 to control for the rate of missed diagnosis in case of the sample size being smaller. The simulation brought good news to us, indeed, that the performance of the estimations was not significantly different between the three kinds of distributions for *p*. It means that, as long as the group of patients comes from an identical population in the sense of statistics, reasonable variation is allowed. In practice, if one recruits a group of patients carefully, following a set of eligibility criteria and exclusion criteria, even though the probabilities of the patients were not being exactly the same, this method still works well, especially for a bigger sample.

In theory, the method developed in this paper could be applied to any other kind of repeated examinations for random responsiveness, not limited to CRS-R for detecting MCS. The fluctuation of the responses of DOC patients to stimuli is closely related to their inherent characteristics. This is not only shown in behavioral assessment but also in other detection and assessment methods (such as functional magnetic resonance imaging assessment technique) (Wang et al., 2015). As Wang et al. (2015) found, there was only a 52% overlap in brain region activation between the two-time functional magnetic resonance imaging tests in DOC patients. Therefore, this method might also be applicable to neuroimaging evaluation. Beyond these, we wish that this method can be further applied to capture some rare event, which is randomly occurring in nature or being detected in a single test.

This paper has some limitations. For the convenience of the readers in the medical field, the abstract mathematical aspect of this method has been avoided, which is intended to be published elsewhere in a professional statistical journal. Since the probabilities of giving a positive response in the problems on MCS patients are possibly in the range of (0.6, 0.9), the stochastic simulation did not cover the range of *p* <0.5. The application on the analysis of two groups of data is just to demonstrate the procedure; further studies with application to patients with different etiology backgrounds need to be explored.

## Data Availability Statement

The original contributions presented in the study are included in the article/Supplementary Material, further inquiries can be directed to the corresponding author/s.

## Ethics Statement

The studies involving human participants were reviewed and approved by the Ethics Committee of Hangzhou Normal University. The patients/participants provided their written informed consent to participate in this study. Written informed consent was obtained from the individual(s) for the publication of any potentially identifiable images or data included in this article.

## Author Contributions

JF and HY designed the study. CY designed the simulation part. HY, XL, LS, and AW collected the data and managed the patients. HY, JW, and NH performed data analyses. JF and HY wrote the draft. JF, OG, SL, and HD revised the manuscript for important academic content. XH took care of the patients. All authors discussed the results, commented on the manuscript, and have read and approved the final manuscript.

## Conflict of Interest

The authors declare that the research was conducted in the absence of any commercial or financial relationships that could be construed as a potential conflict of interest.
