# Corrigendum: Estimating the Minimal Number of Repeated Examinations for Random Responsiveness With the Coma Recovery Scale—Revised as an Example

**DOI:** 10.3389/fnint.2021.745196

**Published:** 2021-09-17

**Authors:** Hao Yang, Chengyin Ye, Xiaochen Liu, Lingxiu Sun, Anqi Wang, Jing Wang, Nantu Hu, Xiaohua Hu, Olivia Gosseries, Steven Laureys, Haibo Di, Jiqian Fang

**Affiliations:** ^1^International Unresponsive Wakefulness Syndrome and Consciousness Science Institute, Hangzhou Normal University, Hangzhou, China; ^2^Department of Health Management, School of Public Health, Hangzhou Normal University, Hangzhou, China; ^3^Hangzhou Wujing Hospital, Hangzhou, China; ^4^Coma Science Group, GIGA- Consciousness and Centre du Cerveau, University and University Hospital of Liège, Liège, Belgium; ^5^School of Public Health, Sun Yat-sen University, Guangzhou, China

**Keywords:** repeated examinations, random responsiveness, diagnosis, Coma Recovery Scale-Revised, disorders of consciousness, minimally conscious state

In the original article, there was a mistake in the legend for Table 5 as published. “${\hat{k}}_{\min}$**” was missed**. The correct legend appears below.

**The numbers of repeated examination**${\hat{k}}_{min}$**for*****p***_***i***_**≡***p*****, *****p***~***N***(***p***, 0.3^2^)and*****p***_***i***_**~***U***(*****p*****−0.3,*****p***+0.3)^*^

In the original article, there was a mistake in Table 1 as published. **The mathematical symbols were misexpressed**. The corrected Table 1 appears below.

**Table 1:** Pooled estimate for the probability of positive response to a single examination given by an MCS patient in theory.

**No. of rounds *i***	**No. of MCSs giving positive response**	**Total no of MCS assessed**.	**Proportion**	**Pooled estimate for the probability of positive response to a single examination**
				**given by an MCS**
1	*a*_1_	*n*	$\frac{a_{1}}{n}$	$\frac{a_{1}}{n}$
2	*a*_2_	*n* − *a*_1_	$\frac{a_{2}}{n-a_{1}}$	$\frac{a_{1}+a_{2}}{n+\left(n-a_{1}\right)}$
3	*a*_3_	*n* − *a*_1_ − *a*_2_	$\frac{a_{3}}{n-a_{1}-a_{2}}$	$\frac{a_{1}+a_{2}+a_{3}}{n+\left(n-a_{1}\right)+\left(n-a_{1}-a_{2}\right)}$
…	…	…	…	…
*i*	*a*_*i*_	*n* − *a*_1_ − … − *a*_*i*−1_	$\frac{a_{i}}{n-a_{1}-\cdots -a_{i-1}}$	$\frac{a_{1}+a_{2}+\cdots +a_{i}}{n+\left(n-a_{1}\right)+\cdots +\left(n-a_{1}-a_{2}-\cdots -a_{i-1}\right)}$

*MCS, minimally conscious state*.

In the original article, there was a mistake in Table 2 as published. **The mathematical symbols were misexpressed**. The corrected Table 2 appears below.

**Table 2:** Estimation for a total number of MCS patients and their probability of giving positive response to a single examination.

**No. of rounds**	**No. of MCSs giving positive response**	**Estimated total no. of MCSs**	**Estimated probability of positive response to a single examination given by an MCS**
***i***	***a*_*i*_**	${\hat{n}}_{i}$	${\hat{p}}_{i}$
1	*a*_1_		
2	*a*_2_	${\hat{n}}_{2}=\frac{a_{1}^{2}}{a_{1}-a_{2}}$	${\hat{p}}_{2}=\frac{a_{1}-a_{2}}{a_{1}}$
3	*a*_3_	${\hat{n}}_{3}=\frac{a_{1}\left(2a_{1}+a_{2}\right)}{2a_{1}-a_{2}-a_{3}}$	${\hat{p}}_{3}=\frac{2a_{1}-a_{2}-a_{3}}{2a_{1}+a_{2}}$
…	…	…	…
*i*	*a*_*i*_	${\hat{n}}_{i}=\frac{a_{1}\left[\left(i-1\right)a_{1}+\left(i-2\right)a_{2}+\cdots +a_{i-1}\right]}{\left(i-1\right)a_{1}-a_{2}-\cdots -a_{i}}$	${\hat{p}}_{i}=\frac{\left(i-1\right)a_{1}-a_{2}-\cdots -a_{i}}{\left(i-1\right)a_{1}+\left(i-2\right)a_{2}+\cdots +a_{i-1}}$

*MCS, minimally conscious state*.

In the original article, there was a mistake in Table 3 as published. **The mathematical symbols were misexpressed**. The corrected Table 3 appears below.

**Table 3:** The data collected from the 13 rounds of successive examinations.

**Group**	**No. of MCSs giving positive response in each round of examinations**													**MCS**	**UWS/VS**	**Total**
	***a*_1_**	***a*_2_**	***a*_3_**	***a*_4_**	***a*_5_**	***a*_6_**	***a*_7_**	***a*_8_**	***a*_9_**	***a*_10_**	***a*_11_**	***a*_12_**	***a*_13_**			
TBI	30	3	3	2	0	0	0	0	0	0	0	0	0	38	12	50
NTBI	29	3	2	1	0	0	0	0	0	0	0	0	0	35	15	50

*MCS, minimally conscious state; UWS/VS, unresponsive wakefulness syndrome/vegetative state; TBI, traumatic brain injury; NTBI, non-traumatic brain injury*.

In the original article, there was some errors. **The mathematical symbols were misexpressed**. A correction has been made to **Materials and methods, Development of statistical formulas**, **Data and formulas, Paragraph 2:**

Since both of *a*_1_/*n* and any of the formulas in the 5-th column of Table 1 approximate to the same probability of positive response to a single examination given by a MCS patient, we have


$$\begin{array}{l} \frac{a_{1}}{n}\approx \frac{a_{1}+a_{2}}{n+\left(n-a_{1}\right)},\\ \frac{a_{1}}{n}\approx \frac{a_{1}+a_{2}+a_{3}}{n+\left(n-a_{1}\right)+\left(n-a_{1}-a_{2}\right)} \end{array}$$

and


$$\begin{array}{l} \frac{a_{1}}{n}\approx \frac{a_{1}+\cdots +a_{i}}{n+\left(n-a_{1}\right)+\cdots \left(n-a_{1}-\cdots -a_{i-1}\right)} \end{array}$$

Denote their solutions of *n*, respectively, by


$$\begin{array}{l} {\hat{n}}_{2}\approx \frac{a_{1}^{2}}{a_{1}-a_{2}},\;{\hat{n}}_{3}\approx \frac{a_{1}\left(2a_{1}+a_{2}\right)}{2a_{1}-a_{2}-a_{3}} \end{array}$$

and


$$\begin{array}{l} {\hat{n}}_{i}\approx \frac{a_{1}\left[\left(i-1\right)a_{1}+\left(i-2\right)a_{2}+\cdots +a_{i-1}\right]}{\left(i-1\right)a_{1}-a_{2}-\cdots -a_{i}},\;{\hat{p}}_{i}=\frac{a_{1}}{{\hat{n}}_{i}} \end{array}$$

These formulas have been summarized in Table 2.

A correction has been made to **Materials and methods, Validation by stochastic simulation**, **“Examination” and “responses,” Paragraph 1:** [0, 1]. **Paragraph 3:** $\hat{n}=\overset{{\hat{k}}_{\min}}{\underset{i=1}{\sum }}a_{i}$, the rate of missed diagnosis $\left(n-\hat{n}\right)/n$.

A correction has been made to **Materials and methods, Validation by stochastic simulation**, **Repeated simulation and the rate of missed diagnosis, Paragraph 1:** $\left(n-\hat{n}\right)/n$.

A correction has been made to **Results, Outcome of Bedside examinations**, **For TBI patients, Paragraph 1–6:**

After completing the first 2 rounds of examinations we obtained the numbers of MCSs giving positive response *a*_1_ = 30 and *a*_2_ = 3, using the formulas in the second row of Table 2, we had the estimated *n* and *p* as


$$\begin{array}{l} {\hat{n}}_{2}\approx \frac{a_{1}^{2}}{a_{1}-a_{2}}=\frac{900}{27}=33.33,\\ {\hat{p}}_{2}\approx \frac{a_{1}}{{\hat{n}}_{2}}=\frac{30}{33.33}=0.9001 \end{array}$$

Since


$$\begin{array}{l} {\left(1-{\hat{p}}_{2}\right)}^{k}\geq 0.0001,k=2,3,4,\\ {\left(1-{\hat{p}}_{2}\right)}^{5}=0.00001<0.0001 \end{array}$$

the examination should be kept on going, and might be ended at the 5-th round; and the total number of MCS patients in this group of DOCs might be around 34.

After completing the 3rd round, we obtained *a*_3_ = 3, and


$$\begin{array}{l} {\hat{n}}_{3}\approx \frac{a_{1}\left(2a_{1}+a_{2}\right)}{2a_{1}-a_{2}-a_{3}}=\frac{30\times 63}{54}=35,\\ {\hat{p}}_{3}\approx \frac{a_{1}}{{\hat{n}}_{3}}=\frac{30}{35}=0.8571 \end{array}$$

Since


$$\begin{array}{l} {\left(1-{\hat{p}}_{3}\right)}^{i}\geq 0.0001,\;i=3,4,\\ {\left(1-{\hat{p}}_{3}\right)}^{5}=0.00006<0.0001 \end{array}$$

the examination should be kept on going, and might be ended at the 5-th round; and the total number of MCS patients in this group of DOCs might be around 35.

After completing the 4-th round, we obtained *a*_4_ = 2, and


$$\begin{array}{l} {\hat{n}}_{4}\approx \frac{a_{1}\left(3a_{1}+2a_{2}+a_{3}\right)}{3a_{1}-a_{2}-a_{3}-a_{4}}=\frac{30\times 99}{82}=36.22,\\ {\hat{p}}_{4}\approx \frac{a_{1}}{{\hat{n}}_{4}}=\frac{30}{36.22}=0.8283 \end{array}$$

Since


$$\begin{array}{l} {\left(1-{\hat{p}}_{4}\right)}^{i}\geq 0.0001,i=4,5,\\ {\left(1-{\hat{p}}_{4}\right)}^{6}=0.00003<0.0001 \end{array}$$

the examination should be kept on going, and might be ended at the 6-th round, and the total number of MCS patients in this group of DOCs might be around 37.

After completing the 5-th round, we obtained *a*_5_ = 0, and


$$\begin{array}{l} {\hat{n}}_{5}\approx \frac{a_{1}\left(4a_{1}+3a_{2}+2a_{3}+a_{4}\right)}{4a_{1}-a_{2}-a_{3}-a_{4}-a_{5}}=\frac{30\times 137}{112}=36.70,\\ {\hat{p}}_{5}\approx \frac{a_{1}}{{\hat{n}}_{5}}=\frac{30}{36.70}=\;0.8175 \end{array}$$

Since


$$\begin{array}{l} {\left(1-{\hat{p}}_{5}\right)}^{5}=0.00020,\\ {\left(1-{\hat{p}}_{5}\right)}^{6}=0.00004<0.0001 \end{array}$$

the examination should be kept on going, and perhaps ended at the 6-th round; and the total number of MCS patients in this group of DOCs might be around 37.

After completing the 6-th round, we obtained *a*_6_ = 0, and


$$\begin{array}{l} {\hat{n}}_{6}\approx \frac{a_{1}\left(5a_{1}+4a_{2}+3a_{3}+2a_{4}{+a}_{5}\right)}{5a_{1}-a_{2}-a_{3}-a_{4}-a_{5}-a_{6}}=\frac{30\times 175}{142}=36.97,\\ {\hat{p}}_{6}\approx \frac{a_{1}}{{\hat{n}}_{6}}=\frac{30}{36.97}=\;0.8114 \end{array}$$

Since


$$\begin{array}{l} {\left(1-{\hat{p}}_{6}\right)}^{6}=0.00005<\;0.0001, \end{array}$$

the examination could be ended at this round ${\hat{k}}_{\text{min}}=6$.

And up to this round, the total number of MCS patients had been detected in the group of DOCs was


$$\begin{array}{l} \hat{n}=a_{1}+a_{2}+a_{3}+a_{4}+a_{5}+a_{6}=38 \end{array}$$

A correction has been made to **Results, Outcome of Bedside examinations**, **For NTBI patients, Paragraph 1–4:**

After completing the first two rounds of examination we obtained the numbers of MCSs giving positive response *a*_1_ = 29 and *a*_2_ = 3, using the formulas in the second row of Table 2, we had


$$\begin{array}{l} {\hat{n}}_{2}=\frac{a_{1}^{2}}{a_{1}-a_{2}}=\frac{29^{2}}{26}=32.35,\\ {\hat{p}}_{2}\approx \frac{a_{1}}{{\hat{n}}_{2}}=\frac{29}{32.35}=0.8966 \end{array}$$

Since


$$\begin{array}{l} {\left(1-{\hat{p}}_{2}\right)}^{i}\geq 0.0001,\;i=2,3,4,\\ {\left(1-{\hat{p}}_{2}\right)}^{5}=0.000012<\;0.0001 \end{array}$$

the examination should be kept on going, and might be ended at the 5-th round; and the total number of MCS patients in this group of DOCs might be 33.

After completing the 3rd round, we obtained *a*_3_ = 2, and


$$\begin{array}{l} {\hat{n}}_{3}=\frac{a_{1}\left(2a_{1}+a_{2}\right)}{2a_{1}-a_{2}-a_{3}}=\frac{29\times 61}{53}=33.38,\\ {\hat{p}}_{3}\approx \frac{a_{1}}{{\hat{n}}_{3}}=\frac{29}{33.38}=\;0.8689 \end{array}$$

Since


$$\begin{array}{l} {\left(1-{\hat{p}}_{3}\right)}^{i}\geq 0.0001,\;i=3,4,\\ {\left(1-{\hat{p}}_{3}\right)}^{5}=0.000039<\;0.0001 \end{array}$$

the examination should be kept on going, and might be ended at the 5-th round; and the total number of MCS patients in this group of DOCs might be 34.

After completing the 4-th round, we obtained *a*_4_ = 1, and


$$\begin{array}{l} {\hat{n}}_{4}=\frac{a_{1}\left(3a_{1}+2a_{2}+a_{3}\right)}{3a_{1}-a_{2}-a_{3}-a_{4}}=\frac{29\times 95}{81}=34.01,\\ {\hat{p}}_{4}\approx \frac{a_{1}}{{\hat{n}}_{4}}=\frac{29}{34.01}=0.8526 \end{array}$$

Since


$$\begin{array}{l} {\left(1-{\hat{p}}_{4}\right)}^{4}=0.000472\geq 0.0001,\\ {\left(1-{\hat{p}}_{4}\right)}^{5}=0.000070<\;0.0001 \end{array}$$

the examination could be ended at 5-th round; and the total number of MCS patients in this group of DOCs might be 35.

After completing the 5-th round, we obtained *a*_5_ = 0, and


$$\begin{array}{l} {\hat{n}}_{5}=\frac{a_{1}\left(4a_{1}+3a_{2}+2a_{3}+a_{4}\right)}{4a_{1}-a_{2}-a_{3}-a_{4}-a_{5}}=\frac{29\times 130}{110}=34.27,\\ {\hat{p}}_{5}\approx \frac{a_{1}}{{\hat{n}}_{5}}=\frac{29}{34.27}=0.8462 \end{array}$$

Since


$$\begin{array}{l} {\left(1-{\hat{p}}_{5}\right)}^{5}=0.000086<0.0001 \end{array}$$

the examination could be ended at this round ${\hat{k}}_{\text{min}}=5$ and up to this round, the total number of MCS patients had been detected in the group of DOCs was


$$\begin{array}{l} \hat{n}=a_{1}+a_{2}+a_{3}+a_{4}+a_{5}=35 \end{array}$$

The authors apologize for the errors and state that this does not change the scientific conclusions of the article in any way. The original article has been updated.

## Publisher's Note

All claims expressed in this article are solely those of the authors and do not necessarily represent those of their affiliated organizations, or those of the publisher, the editors and the reviewers. Any product that may be evaluated in this article, or claim that may be made by its manufacturer, is not guaranteed or endorsed by the publisher.

