# Supplementary material for: Estimating the Minimal Number of Repeated Examinations for Random Responsiveness With the Coma Recovery Scale—Revised as an Example
Source: Front Integr Neurosci. 2021 Jul 8;15:685627. doi: 10.3389/fnint.2021.685627 (PMC8297543; doi:10.3389/fnint.2021.685627)
Supplement: Supplementary file 1 [file Data_Sheet_1.docx]

**Supplementary Material A：**

**Table A The characteristic data of 100 disorders of consciousness patients**

| Patients | Sex | Age（years） | Cause | TSI months | CRS-R (best) | CRS-R total score | Diagnosis | Number of examinations |
| --- | --- | --- | --- | --- | --- | --- | --- | --- |
| 1 | M | 74 | TBI | 8 | 2-3-5-1-0-2 | 13 | MCS- | 1 |
| 2 | F | 65 | TBI | 3.7 | 0-0-5-1-0-2 | 8 | MCS- | 1 |
| 3 | F | 71 | TBI | 10 | 2-2-5-2-0-2 | 13 | MCS- | 1 |
| 4 | M | 32 | TBI | 4.9 | 2-3-2-1-0-2 | 10 | MCS- | 1 |
| 5 | M | 64 | TBI | 3.2 | 2-2-5-2-0-2 | 13 | MCS- | 1 |
| 6 | M | 37 | TBI | 9.8 | 1-3-2-1-0-1 | 8 | MCS- | 1 |
| 7 | M | 73 | TBI | 1.4 | 0-0-5-0-0-2 | 7 | MCS- | 1 |
| 8 | F | 47 | TBI | 5.3 | 1-1-5-1-0-1 | 9 | MCS- | 1 |
| 9 | M | 67 | TBI | 10.2 | 1-3-5-2-0-2 | 13 | MCS- | 1 |
| 10 | M | 71 | TBI | 1.1 | 0-1-5-1-0-1 | 8 | MCS- | 1 |
| 11 | F | 61 | TBI | 2.3 | 2-4-5-2-0-2 | 15 | MCS- | 1 |
| 12 | M | 73 | TBI | 1.2 | 0-1-5-1-0-1 | 8 | MCS- | 1 |
| 13 | M | 59 | TBI | 14 | 1-4-2-1-0-1 | 9 | MCS- | 1 |
| 14 | M | 65 | TBI | 7 | 1-3-2-1-0-1 | 8 | MCS- | 1 |
| 15 | F | 18 | TBI | 5 | 1-3-3-1-0-2 | 10 | MCS- | 1 |
| 16 | M | 71 | TBI | 5.5 | 0-1-3-1-0-2 | 7 | MCS- | 1 |
| 17 | M | 59 | TBI | 2 | 0-1-3-0-0-1 | 5 | MCS- | 1 |
| 18 | M | 66 | TBI | 1.5 | 1-1-3-0-0-1 | 6 | MCS- | 1 |
| 19 | M | 52 | TBI | 1.5 | 0-1-5-0-0-2 | 8 | MCS- | 1 |
| 20 | M | 43 | TBI | 12 | 0-0-3-1-0-0 | 4 | MCS- | 1 |
| 21 | M | 46 | TBI | 24 | 1-2-5-1-0-2 | 11 | MCS- | 1 |
| 22 | M | 51 | TBI | 5.1 | 0-0-5-0-0-1 | 6 | MCS- | 1 |
| 23 | M | 56 | TBI | 9.4 | 1-3-5-1-0-1 | 11 | MCS- | 1 |
| 24 | M | 54 | TBI | 6 | 0-1-5-1-0-2 | 9 | MCS- | 1 |
| 25 | M | 38 | TBI | 3.3 | 2-3-5-2-0-2 | 14 | MCS- | 1 |
| 26 | M | 38 | TBI | 2.3 | 3-4-5-1-0-1 | 14 | MCS+ | 1 |
| 27 | M | 58 | TBI | 6 | 2-3-5-3-1-1 | 15 | MCS+ | 1 |
| 28 | M | 56 | TBI | 2.5 | 3-4-5-1-0-2 | 15 | MCS+ | 1 |
| Patients | Sex | Age（years） | Cause | TSI months | CRS-R (best) | CRS-R total score | Diagnosis | Number of examinations |
| 29 | M | 68 | TBI | 1.5 | 2-4-5-3-0-1 | 15 | MCS+ | 1 |
| 30 | M | 62 | TBI | 1 | 2-4-5-3-1-3 | 18 | MCS+ | 1 |
| 31 | M | 77 | TBI | 9.2 | 0-4-5-1-0-1 | 11 | MCS- | 2 |
| 32 | M | 45 | TBI | 11.2 | 0-0-5-1-0-1 | 7 | MCS- | 2 |
| 33 | M | 68 | TBI | 7.5 | 1-3-2-1-0-1 | 8 | MCS- | 2 |
| 34 | M | 58 | TBI | 5.5 | 0-3-2-1-0-2 | 8 | MCS- | 3 |
| 35 | M | 44 | TBI | 1.5 | 1-2-2-1-0-2 | 8 | MCS- | 3 |
| 36 | F | 48 | TBI | 1.5 | 2-3-1-0-0-2 | 8 | MCS- | 3 |
| 37 | M | 73 | TBI | 4.1 | 1-1-4-1-0-1 | 8 | MCS- | 4 |
| 38 | F | 75 | TBI | 8 | 1-3-2-0-0-2 | 8 | MCS- | 4 |
| 39 | F | 64 | TBI | 12.5 | 0-1-2-1-0-2 | 6 | UWS | 13 |
| 40 | F | 69 | TBI | 9 | 1-1-2-1-0-2 | 7 | UWS | 13 |
| 41 | M | 59 | TBI | 1.9 | 1-1-2-1-0-2 | 7 | UWS | 13 |
| 42 | F | 62 | TBI | 8.2 | 0-0-2-1-0-2 | 5 | UWS | 13 |
| 43 | M | 48 | TBI | 6.3 | 1-1-2-1-0-2 | 7 | UWS | 13 |
| 44 | M | 38 | TBI | 3.2 | 1-1-2-1-0-1 | 6 | UWS | 13 |
| 45 | M | 71 | TBI | 3 | 1-1-2-1-0-2 | 7 | UWS | 13 |
| 46 | M | 51 | TBI | 2 | 1-1-2-1-0-1 | 6 | UWS | 13 |
| 47 | M | 57 | TBI | 2 | 0-1-2-1-0-1 | 5 | UWS | 13 |
| 48 | M | 69 | TBI | 6 | 0-1-2-0-0-2 | 5 | UWS | 13 |
| 49 | M | 70 | TBI | 9.5 | 1-0-2-2-0-2 | 7 | UWS | 13 |
| 50 | M | 56 | TBI | 2.4 | 1-0-2-1-0-2 | 6 | UWS | 13 |
| 51 | F | 48 | NTBI | 5 | 0-3-5-2-0-2 | 12 | MCS- | 1 |
| 52 | M | 44 | NTBI | 17.5 | 0-3-2-1-0-2 | 8 | MCS- | 1 |
| 53 | M | 83 | NTBI | 1 | 0-4-4-1-0-2 | 11 | MCS- | 1 |
| 54 | F | 71 | NTBI | 1.5 | 1-3-2-1-0-2 | 9 | MCS- | 1 |
| 55 | F | 58 | NTBI | 5 | 1-3-2-1-0-1 | 8 | MCS- | 1 |
| 56 | M | 58 | NTBI | 10 | 0-4-5-2-0-2 | 13 | MCS- | 1 |
| 57 | M | 43 | NTBI | 1.5 | 0-0-3-1-0-1 | 5 | MCS- | 1 |
| 58 | M | 69 | NTBI | 3.5 | 0-0-3-1-0-0 | 4 | MCS- | 1 |
| Patients | Sex | Age（years） | Cause | TSI months | CRS-R (best) | CRS-R total score | Diagnosis | Number of examinations |
| 59 | M | 62 | NTBI | 1 | 1-3-3-1-0-0 | 8 | MCS- | 1 |
| 60 | M | 55 | NTBI | 4.5 | 1-1-3-1-0-2 | 8 | MCS- | 1 |
| 61 | M | 42 | NTBI | 3.2 | 0-3-2-0-0-1 | 6 | MCS- | 1 |
| 62 | F | 53 | NTBI | 27.8 | 1-2-3-1-0-2 | 9 | MCS- | 1 |
| 63 | M | 68 | NTBI | 8.7 | 2-3-5-0-0-2 | 12 | MCS- | 1 |
| 64 | M | 38 | NTBI | 2 | 3-5-5-2-1-2 | 18 | MCS+ | 1 |
| 65 | M | 41 | NTBI | 2.5 | 4-5-5-3-1-3 | 21 | MCS+ | 1 |
| 66 | M | 70 | NTBI | 12.7 | 3-4-5-1-0-2 | 15 | MCS+ | 1 |
| 67 | F | 68 | NTBI | 1.1 | 3-1-3-2-1-2 | 12 | MCS+ | 1 |
| 68 | F | 66 | NTBI | 5 | 3-4-4-0-0-2 | 13 | MCS+ | 1 |
| 69 | F | 65 | NTBI | 2.6 | 1-3-2-1-0-1 | 8 | MCS- | 1 |
| 70 | M | 71 | NTBI | 1.5 | 2-3-2-1-0-1 | 9 | MCS- | 1 |
| 71 | M | 82 | NTBI | 1.3 | 0-1-5-0-0-1 | 7 | MCS- | 1 |
| 72 | M | 38 | NTBI | 1 | 2-3-5-0-0-2 | 12 | MCS- | 1 |
| 73 | M | 77 | NTBI | 1 | 2-4-5-2-0-3 | 16 | MCS- | 1 |
| 74 | M | 79 | NTBI | 5.8 | 1-3-2-1-0-2 | 9 | MCS- | 1 |
| 75 | M | 78 | NTBI | 4 | 3-3-0-0-1-2 | 9 | MCS+ | 1 |
| 76 | M | 50 | NTBI | 1.7 | 3-5-5-0-1-3 | 13 | MCS+ | 1 |
| 77 | F | 83 | NTBI | 1 | 3-5-5-0-1-3 | 17 | MCS+ | 1 |
| 78 | F | 70 | NTBI | 3.2 | 3-4-5-2-1-2 | 17 | MCS+ | 1 |
| 79 | F | 49 | NTBI | 2.3 | 2-4-5-1-0-1 | 13 | MCS- | 1 |
| 80 | M | 54 | NTBI | 6.3 | 2-3-2-1-0-1 | 9 | MCS- | 2 |
| 81 | M | 74 | NTBI | 2.6 | 1-4-2-1-0-1 | 9 | MCS- | 2 |
| 82 | M | 63 | NTBI | 4.5 | 1-3-1-0-0-1 | 6 | MCS- | 2 |
| 83 | M | 42 | NTBI | 3 | 0-2-1-0-0-2 | 5 | MCS- | 3 |
| 84 | M | 63 | NTBI | 13.1 | 1-3-1-0-0-2 | 7 | MCS- | 3 |
| 85 | M | 55 | NTBI | 7.3 | 1-3-2-1-0-1 | 8 | MCS- | 4 |
| 86 | M | 63 | NTBI | 4 | 1-0-1-1-0-1 | 4 | UWS | 13 |
| 87 | M | 78 | NTBI | 1.5 | 1-1-2-1-0-2 | 7 | UWS | 13 |
| 88 | F | 67 | NTBI | 1 | 1-1-2-1-0-1 | 6 | UWS | 13 |
| Patients | Sex | Age（years） | Cause | TSI months | CRS-R (best) | CRS-R total score | Diagnosis | Number of examinations |
| 89 | F | 48 | NTBI | 3.5 | 1-1-2-1-0-2 | 7 | UWS | 13 |
| 90 | M | 69 | NTBI | 4 | 1-1-2-1-0-2 | 7 | UWS | 13 |
| 91 | M | 39 | NTBI | 2 | 1-1-2-1-0-2 | 7 | UWS | 13 |
| 92 | F | 56 | NTBI | 2.6 | 0-0-2-1-0-1 | 4 | UWS | 13 |
| 93 | F | 43 | NTBI | 4.7 | 1-0-2-1-0-2 | 6 | UWS | 13 |
| 94 | M | 61 | NTBI | 1.5 | 1-1-2-1-0-1 | 6 | UWS | 13 |
| 95 | M | 78 | NTBI | 5.3 | 0-0-2-1-0-1 | 4 | UWS | 13 |
| 96 | M | 34 | NTBI | 3.9 | 1-1-2-1-0-2 | 7 | UWS | 13 |
| 97 | M | 30 | NTBI | 2.8 | 1-1-2-1-0-2 | 7 | UWS | 13 |
| 98 | F | 26 | NTBI | 2.5 | 1-1-2-1-0-2 | 7 | UWS | 13 |
| 99 | M | 68 | NTBI | 5.5 | 1-0-2-2-0-2 | 7 | UWS | 13 |
| 100 | F | 63 | NTBI | 1 | 1-0-2-1-0-1 | 5 | UWS | 13 |

*TSI: time since injury (months after injury); CRS-R: Coma Recovery Scale-Revised; M: male; F: female; TBI: traumatic brain injury; NTBI: non- traumatic brain injury, UWS*: *unresponsive wakefulness syndrome; MCS-: minimally conscious state minus; MCS+: minimally conscious state plus.*

**Supplementary Material B：**

We developed a general method for estimation of the minimal number of repeated examinations needed to detect patients with random responsiveness given a limited rate of missed diagnosis. To validate this method, a series of stochastic simulations has been carried out using R programming under all possible combinations of 3 types of distributions for $p_{i}$ (constant, normal and uniform), 5 values of $p$ ($p$ = 0.5, 0.6, 0.8, 0.9) and 4 sizes of sample ($n$= 50, 100, 500, 1000), with each scenario repeated for 100 times.

Table B1.1 lists the results of 100 replicates under the scenario of constant $p_{i}$=0.7 and sample size 1000. Table B1.2 shows the mean and 95% confidence interval (CI) of the estimates corresponding to different sample sizes and constant $p_{i}$=0.7. Table B1.3 lists the detailed results under the scenario of $p_{i}\sim N(0.7,0.1^{2})$ and sample size 1000. Table B1.4 shows the mean and 95% CI of the estimates corresponding to different sample sizes and$p_{i}\sim N(0.7,0.1^{2})$. Table B1.5 lists the results of 100 replicates under the scenario of$p_{i}\sim N(0.7,0.03^{2})$, and sample size 1000. Table B1.6 shows the mean and 95% CI of the estimates corresponding to different sample sizes and $p_{i}\sim N(0.7,0.03^{2})$. Table B1.7 lists the results of 100 replicates under the scenario of $p_{i}\sim U(0.69,0.71)$, and sample size 1000. Table B1.8 shows the mean and 95% CI of the estimates corresponding to different sample sizes and$p_{i}\sim U(0.69,0.71)$. Table B1.9 lists the results of 100 replicates under the scenario of $p_{i}\sim U(0.67,0.73)$ and sample size 1000. Table B1.10 shows the mean and 95% CI of the estimates corresponding to different sample sizes and $p_{i}\sim U(0.67,0.73)$.

Table B2.1 to Table B2.4 list the mean and 95% CI of the estimates corresponding to different conditions of constant *p* (0.5, 0.6, 0.7, 0.8, 0.9) and sample sizes ($n$= 50, 100, 500, 1000).

Table B3.1 to Table B3.4 list the mean and 95% CI of the estimates corresponding to different conditions of $p_{i}\sim N\left( p,0.1^{2} \right), p=0.5, 0.6, 0.7, 0.8, 0.9$.

Table B4.1 to Table B4.5 list the mean and 95% CI of the estimates corresponding to different conditions of $p_{i}\sim N\left( p,0.2^{2} \right), p=0.5, 0.6, 0.7, 0.8, 0.9$.

Table B5.1 to Table B5.4 list the mean and 95% CI of the estimates corresponding to different conditions of $p_{i}\sim N\left( p,0.3^{2} \right), p=0.5, 0.6, 0.7, 0.8, 0.9$.

Table B6.1 to Table B6.4 list the mean and 95% CI of the estimates corresponding to different conditions of $p_{i}\sim U\left( p-0.1,p+0.1 \right), p=0.5, 0.6, 0.7, 0.8, 0.9$.

Table B7.1 to Table B7.5 lists the mean and 95% CI of the estimates for 5 different conditions of uniform distributed $p_{i}\sim U\left( p-0.2,p+0.2 \right), p=0.5, 0.6, 0.7, 0.8, 0.9$.

Table B8.1 to Table B8.4 lists the mean and 95% CI of the estimates for 4 different conditions of uniform distributed $p_{i}\sim U\left( p-0.3,p+0.3 \right), p=0.5, 0.6, 0.7, 0.8, 0.9$.

**Table B1.1 Summary for** $\boldsymbol{p}_{\boldsymbol{i}}\boldsymbol{=0.7}$**, *n* =1000**

| **ID** | $\boldsymbol{a}_{\boldsymbol{1}}$ | $\boldsymbol{a}_{\boldsymbol{2}}$ | $\boldsymbol{a}_{\boldsymbol{3}}$ | $\boldsymbol{a}_{\boldsymbol{4}}$ | $\boldsymbol{a}_{\boldsymbol{5}}$ | $\boldsymbol{a}_{\boldsymbol{6}}$ | $\boldsymbol{a}_{\boldsymbol{7}}$ | $\boldsymbol{a}_{\boldsymbol{8}}$ | $\boldsymbol{a}_{\boldsymbol{9}}$ | $\hat{\boldsymbol{n}}$**^*^** | $\hat{\boldsymbol{p}}$**^#^** | ${\hat{\boldsymbol{k}}}_{\text{min}}$ | $\boldsymbol{n}\boldsymbol{-}\hat{\boldsymbol{n}}$ |
| --- | --- | --- | --- | --- | --- | --- | --- | --- | --- | --- | --- | --- | --- |
| **1** | 699 | 205 | 73 | 13 | 6 | 3 | 1 | 0 | - | 1000 | 0.70 | 8 | 0 |
| **2** | 719 | 192 | 61 | 19 | 7 | 2 | 0 | 0 | - | 1000 | 0.72 | 8 | 0 |
| **3** | 709 | 208 | 64 | 15 | 2 | 1 | 0 | 0 | - | 999 | 0.71 | 8 | 1 |
| **4** | 715 | 194 | 71 | 15 | 3 | 2 | 0 | 0 | - | 1000 | 0.72 | 8 | 0 |
| **…** | … | … | … | … | … | … | … | … | … | … | … | … | … |
| **25** | 708 | 186 | 72 | 22 | 11 | 1 | 0 | 0 | - | 1000 | 0.71 | 8 | 0 |
| **26** | 699 | 194 | 78 | 18 | 6 | 3 | 0 | 0 | - | 998 | 0.70 | 8 | 2 |
| **27** | 700 | 216 | 58 | 18 | 8 | 0 | 0 | 0 | - | 1000 | 0.70 | 8 | 0 |
| **…** | … | … | … | … | … | … | … | … | … | … | … | … | … |
| **34** | 686 | 217 | 63 | 21 | 7 | 5 | 1 | 0 | - | 1000 | 0.69 | 8 | 0 |
| **35** | 716 | 197 | 61 | 17 | 4 | 1 | 3 | 0 | - | 999 | 0.72 | 8 | 1 |
| **36** | 700 | 211 | 57 | 20 | 7 | 2 | 3 | 0 | - | 1000 | 0.70 | 8 | 0 |
| **…** | … | … | … | … | … | … | … | … | … | … | … | … | … |
| **40** | 720 | 188 | 60 | 19 | 10 | 2 | 1 | 0 | - | 1000 | 0.72 | 8 | 0 |
| **41** | 691 | 218 | 69 | 15 | 5 | 0 | 1 | 0 | - | 999 | 0.69 | 8 | 1 |
| **42** | 670 | 235 | 66 | 23 | 5 | 1 | 0 | 0 | 0 | 1000 | 0.67 | 9 | 0 |
| **…** | … | … | … | … | … | … | … | … | … | … | … | … | … |
| **48** | 705 | 203 | 69 | 17 | 4 | 2 | 0 | 0 | - | 1000 | 0.71 | 8 | 0 |
| **ID** | $\boldsymbol{a}_{\boldsymbol{1}}$ | $\boldsymbol{a}_{\boldsymbol{2}}$ | $\boldsymbol{a}_{\boldsymbol{3}}$ | $\boldsymbol{a}_{\boldsymbol{4}}$ | $\boldsymbol{a}_{\boldsymbol{5}}$ | $\boldsymbol{a}_{\boldsymbol{6}}$ | $\boldsymbol{a}_{\boldsymbol{7}}$ | $\boldsymbol{a}_{\boldsymbol{8}}$ | $\boldsymbol{a}_{\boldsymbol{9}}$ | $\hat{\boldsymbol{n}}$**^*^** | $\hat{\boldsymbol{p}}$**^#^** | ${\hat{\boldsymbol{k}}}_{\text{min}}$ | $\boldsymbol{n}\boldsymbol{-}\hat{\boldsymbol{n}}$ |
| **49** | 701 | 220 | 55 | 21 | 2 | 0 | 0 | 0 | - | 999 | 0.70 | 8 | 1 |
| **50** | 670 | 245 | 57 | 23 | 4 | 1 | 0 | 0 | 0 | 1000 | 0.67 | 9 | 0 |
| **…** | … | … | … | … | … | … | … | … | … | … | … | … | … |
| **58** | 705 | 206 | 61 | 19 | 4 | 2 | 3 | 0 | - | 1000 | 0.71 | 8 | 0 |
| **59** | 707 | 190 | 63 | 28 | 8 | 1 | 1 | 1 | - | 999 | 0.71 | 8 | 1 |
| **60** | 696 | 216 | 66 | 17 | 3 | 1 | 1 | 0 | - | 1000 | 0.70 | 8 | 0 |
| **…** | … | … | … | … | … | … | … | … | … | … | … | … | … |
| **71** | 704 | 204 | 62 | 19 | 8 | 2 | 0 | 1 | - | 1000 | 0.70 | 8 | 0 |
| **72** | 698 | 214 | 60 | 20 | 7 | 0 | 0 | 0 | - | 999 | 0.70 | 8 | 1 |
| **73** | 689 | 220 | 69 | 14 | 8 | 0 | 0 | 0 | - | 1000 | 0.69 | 8 | 0 |
| **…** | … | … | … | … | … | … | … | … | … | … | … | … | … |
| **100** | 701 | 207 | 58 | 26 | 6 | 2 | 0 | 0 | - | 1000 | 0.70 | 8 | 0 |
| **Mean** | 698.96 | 210.14 | 63.86 | 18.97 | 5.78 | 1.51 | 0.51 | 0.18 | 0.07 | 999.92 | 0.70 | 8.14 | 0.08 |

**^*^**$\hat{\boldsymbol{n}}\boldsymbol{=}\boldsymbol{a}_{\boldsymbol{1}}\boldsymbol{+}\boldsymbol{a}_{\boldsymbol{2}}\boldsymbol{+\cdots}\boldsymbol{a}_{\boldsymbol{k}_{\text{mini}}}$**; ^#^**$\hat{\boldsymbol{p}}\boldsymbol{=}\boldsymbol{a}_{\boldsymbol{1}}\boldsymbol{/}\hat{\boldsymbol{n}}$

**Table B1.2 Summary for** $\boldsymbol{p}_{\boldsymbol{i}}\boldsymbol{=0.7}$**, *n* =1000, 500, 100, 5**

|  | | **Mean and 95% Confidence interval** | | | | |
| --- | --- | --- | --- | --- | --- | --- |
| $\boldsymbol{n}$ | $\hat{\boldsymbol{n}}$ | | $\hat{\boldsymbol{p}}$ | ${\hat{\boldsymbol{k}}}_{\text{min}}$ | $\boldsymbol{n}\boldsymbol{-}\hat{\boldsymbol{n}}$ | $\frac{\boldsymbol{n}\boldsymbol{-}\hat{\boldsymbol{n}}}{\boldsymbol{n}}$ |
| **1000** | 999.92±0.06 | | 0.70±0.003 | 8.14±0.07 | 0.08±0.06 | 0.00008±0.00006 |
| **500** | 499.96±0.04 | | 0.70±0.004 | 8.13±0.10 | 0.04±0.04 | 0.00008±0.00008 |
| **100** | 99.99±0.02 | | 0.71±0.009 | 7.96±0.21 | 0.01±0.02 | 0.00010±0.00020 |
| **50** | 50 | | 0.71±0.011 | 8.11±0.28 | 0 | 0 |

**Table B1.3 Summary for** $\boldsymbol{p}_{\boldsymbol{i}}\boldsymbol{\sim N(0.7,0.0}\boldsymbol{1}^{\boldsymbol{2}}\boldsymbol{)}$**, *n* =1000**

| **ID** | $\boldsymbol{a}_{\boldsymbol{1}}$ | $\boldsymbol{a}_{\boldsymbol{2}}$ | $\boldsymbol{a}_{\boldsymbol{3}}$ | $\boldsymbol{a}_{\boldsymbol{4}}$ | $\boldsymbol{a}_{\boldsymbol{5}}$ | $\boldsymbol{a}_{\boldsymbol{6}}$ | $\boldsymbol{a}_{\boldsymbol{7}}$ | $\boldsymbol{a}_{\boldsymbol{8}}$ | $\boldsymbol{a}_{\boldsymbol{9}}$ | $\hat{\boldsymbol{n}}$**^*^** | $\hat{\boldsymbol{p}}$**^#^** | ${\hat{\boldsymbol{k}}}_{\text{min}}$ | $\boldsymbol{n}\boldsymbol{-}\hat{\boldsymbol{n}}$ |
| --- | --- | --- | --- | --- | --- | --- | --- | --- | --- | --- | --- | --- | --- |
| **1** | 718 | 187 | 71 | 18 | 4 | 2 | 0 | 0 | - | 1000 | 0.72 | 8 | 0 |
| **2** | 704 | 191 | 68 | 24 | 10 | 2 | 0 | 1 | - | 1000 | 0.70 | 8 | 0 |
| **3** | 684 | 205 | 87 | 20 | 3 | 1 | 0 | 0 | 0 | 1000 | 0.68 | 9 | 0 |
| **…** | … | … | … | … | … | … | … | … | … | … | … | … | … |
| **14** | 688 | 209 | 71 | 24 | 7 | 1 | 0 | 0 | - | 1000 | 0.69 | 8 | 0 |
| **15** | 697 | 219 | 53 | 19 | 5 | 5 | 1 | 0 | - | 999 | 0.70 | 8 | 1 |
| **16** | 681 | 223 | 67 | 20 | 6 | 1 | 0 | 0 | 2 | 1000 | 0.68 | 9 | 0 |
| **…** | … | … | … | … | … | … | … | … | … | … | … | … | … |
| **27** | 720 | 197 | 55 | 17 | 9 | 2 | 0 | 0 | - | 1000 | 0.72 | 8 | 0 |
| **28** | 708 | 196 | 62 | 21 | 6 | 5 | 1 | 0 | - | 999 | 0.71 | 8 | 1 |
| **29** | 703 | 206 | 62 | 17 | 7 | 5 | 0 | 0 | - | 1000 | 0.70 | 8 | 0 |
| **30** | 704 | 210 | 61 | 18 | 4 | 1 | 2 | 0 | - | 1000 | 0.70 | 8 | 0 |
| **31** | 684 | 223 | 69 | 19 | 3 | 1 | 0 | 0 | 0 | 999 | 0.68 | 9 | 1 |
| **32** | 707 | 214 | 54 | 17 | 6 | 2 | 0 | 0 | - | 1000 | 0.71 | 8 | 0 |
| **33** | 697 | 213 | 60 | 17 | 9 | 4 | 0 | 0 | - | 1000 | 0.70 | 8 | 0 |
| **34** | 678 | 217 | 69 | 25 | 7 | 3 | 0 | 0 | 0 | 999 | 0.68 | 9 | 1 |
| **…** | … | … | … | … | … | … | … | … | … | … | … | … | … |
| **49** | 693 | 207 | 75 | 20 | 4 | 1 | 0 | 0 | - | 1000 | 0.69 | 8 | 0 |
| **ID** | $\boldsymbol{a}_{\boldsymbol{1}}$ | $\boldsymbol{a}_{\boldsymbol{2}}$ | $\boldsymbol{a}_{\boldsymbol{3}}$ | $\boldsymbol{a}_{\boldsymbol{4}}$ | $\boldsymbol{a}_{\boldsymbol{5}}$ | $\boldsymbol{a}_{\boldsymbol{6}}$ | $\boldsymbol{a}_{\boldsymbol{7}}$ | $\boldsymbol{a}_{\boldsymbol{8}}$ | $\boldsymbol{a}_{\boldsymbol{9}}$ | $\hat{\boldsymbol{n}}$**^*^** | $\hat{\boldsymbol{p}}$**^#^** | ${\hat{\boldsymbol{k}}}_{\text{min}}$ | $\boldsymbol{n}\boldsymbol{-}\hat{\boldsymbol{n}}$ |
| **50** | 703 | 209 | 65 | 14 | 3 | 4 | 0 | 0 | - | 998 | 0.70 | 8 | 2 |
| **51** | 711 | 198 | 65 | 23 | 1 | 0 | 2 | 0 | - | 1000 | 0.71 | 8 | 0 |
| **…** | … | … | … | … | … | … | … | … | … | … | … | … | … |
| **90** | 670 | 245 | 55 | 19 | 8 | 1 | 2 | 0 | 0 | 1000 | 0.67 | 9 | 0 |
| **91** | 694 | 220 | 54 | 21 | 7 | 2 | 0 | 1 | - | 999 | 0.69 | 8 | 1 |
| **92** | 712 | 210 | 47 | 24 | 6 | 1 | 0 | 0 | - | 1000 | 0.71 | 8 | 0 |
| **93** | 702 | 190 | 77 | 21 | 4 | 3 | 2 | 0 | - | 999 | 0.70 | 8 | 1 |
| **94** | 688 | 224 | 60 | 23 | 4 | 1 | 0 | 0 | - | 1000 | 0.69 | 8 | 0 |
| **…** | … | … | … | … | … | … | … | … | … | … | … | … | … |
| **100** | 688 | 211 | 71 | 19 | 9 | 1 | 0 | 1 | **-** | 1000 | 0.69 | 8 | 0 |
| **Mean** | 700.12 | 208.32 | 63.88 | 19.21 | 5.94 | 1.85 | 0.43 | 0.13 | 0.36 | 999.92 | 0.70 | 8.08 | 0.08 |

**^*^**$\hat{\boldsymbol{n}}\boldsymbol{=}\boldsymbol{a}_{\boldsymbol{1}}\boldsymbol{+}\boldsymbol{a}_{\boldsymbol{2}}\boldsymbol{+}\boldsymbol{\cdots}\boldsymbol{a}_{\boldsymbol{k}_{\text{mini}}}$**; ^#^**$\hat{\boldsymbol{p}}\boldsymbol{=}\boldsymbol{a}_{\boldsymbol{1}}\boldsymbol{/}\hat{\boldsymbol{n}}$

| **Table B1.4 Summary for** $\boldsymbol{p}_{\boldsymbol{i}}\boldsymbol{\sim N(0.7,0.0}\boldsymbol{1}^{\boldsymbol{2}}\boldsymbol{)}$**, *n* =1000, 500, 100, 50** | | | | | |
| --- | --- | --- | --- | --- | --- |
|  | **Mean and 95% Confidence interval** | | | | |
| $\boldsymbol{n}$ | $\hat{\boldsymbol{n}}$ | $\hat{\boldsymbol{p}}$ | ${\hat{\boldsymbol{k}}}_{\text{min}}$ | $\boldsymbol{n}\boldsymbol{-}\hat{\boldsymbol{n}}$ | $\frac{\boldsymbol{n}\boldsymbol{-}\hat{\boldsymbol{n}}}{\boldsymbol{n}}$ |
| **1000** | 999.92±0.06 | 0.70±0.003 | 8.08±0.07 | 0.08±0.06 | 0.00008±0.00006 |
| **500** | 499.98±0.03 | 0.70±0.004 | 8.10±0.10 | 0.02±0.03 | 0.00004±0.00006 |
| **100** | 99.98±0.03 | 0.70±0.009 | 8.13±0.22 | 0.02±0.03 | 0.00020±0.00028 |
| **50** | 50 | 0.70±0.013 | 8.28±0.31 | 0 | 0 |

**Table B1.5 Summary for** $\boldsymbol{p}_{\boldsymbol{i}}\boldsymbol{\sim N(0.7,0.0}3^{\boldsymbol{2}}\boldsymbol{)}$**, *n* =1000**

| **ID** | $\boldsymbol{a}_{\boldsymbol{1}}$ | $\boldsymbol{a}_{\boldsymbol{2}}$ | $\boldsymbol{a}_{\boldsymbol{3}}$ | $\boldsymbol{a}_{\boldsymbol{4}}$ | $\boldsymbol{a}_{\boldsymbol{5}}$ | $\boldsymbol{a}_{\boldsymbol{6}}$ | $\boldsymbol{a}_{\boldsymbol{7}}$ | $\boldsymbol{a}_{\boldsymbol{8}}$ | $\boldsymbol{a}_{\boldsymbol{9}}$ | $\hat{\boldsymbol{n}}$**^*^** | $\hat{\boldsymbol{p}}$**^#^** | ${\hat{\boldsymbol{k}}}_{\text{min}}$ | $\boldsymbol{n}\boldsymbol{-}\hat{\boldsymbol{n}}$ |
| --- | --- | --- | --- | --- | --- | --- | --- | --- | --- | --- | --- | --- | --- |
| **1** | 711 | 189 | 74 | 19 | 3 | 3 | 1 | 0 | - | 1000 | 0.711 | 8 | 0 |
| **2** | 698 | 190 | 75 | 24 | 8 | 3 | 0 | 2 | - | 1000 | 0.698 | 8 | 0 |
| **3** | 686 | 214 | 79 | 17 | 4 | 0 | 0 | 0 | - | 1000 | 0.686 | 8 | 0 |
| **…** | … | … | … | … | … | … | … | … | … | … | … | … | … |
| **24** | 716 | 205 | 57 | 14 | 5 | 3 | 0 | 0 | - | 1000 | 0.716 | 8 | 0 |
| **25** | 690 | 219 | 69 | 15 | 4 | 1 | 0 | 1 | - | 999 | 0.691 | 8 | 1 |
| **26** | 712 | 185 | 76 | 19 | 5 | 2 | 1 | 0 | - | 1000 | 0.712 | 8 | 0 |
| **27** | 725 | 190 | 55 | 19 | 7 | 3 | 0 | 0 | - | 999 | 0.726 | 8 | 1 |
| **28** | 706 | 198 | 63 | 23 | 9 | 1 | 0 | 0 | - | 1000 | 0.706 | 8 | 0 |
| **…** | … | … | … | … | … | … | … | … | … | … | … | … | … |
| **37** | 691 | 214 | 63 | 22 | 8 | 0 | 2 | 0 | - | 1000 | 0.691 | 8 | 0 |
| **38** | 685 | 205 | 82 | 21 | 5 | 1 | 0 | 0 | - | 999 | 0.686 | 8 | 1 |
| **39** | 693 | 211 | 65 | 22 | 4 | 4 | 1 | 0 | - | 1000 | 0.693 | 8 | 0 |
| **40** | 684 | 222 | 65 | 23 | 5 | 1 | 0 | 0 | 0 | 1000 | 0.684 | 9 | 0 |
| **41** | 720 | 186 | 67 | 16 | 7 | 2 | 0 | 1 | - | 999 | 0.721 | 8 | 1 |
| **42** | 700 | 208 | 63 | 18 | 6 | 2 | 1 | 1 | - | 999 | 0.701 | 8 | 1 |
| **43** | 693 | 214 | 72 | 13 | 4 | 3 | 1 | 0 | - | 1000 | 0.693 | 8 | 0 |
| **…** | … | … | … | … | … | … | … | … | … | … | … | … | … |
| **ID** | $\boldsymbol{a}_{\boldsymbol{1}}$ | $\boldsymbol{a}_{\boldsymbol{2}}$ | $\boldsymbol{a}_{\boldsymbol{3}}$ | $\boldsymbol{a}_{\boldsymbol{4}}$ | $\boldsymbol{a}_{\boldsymbol{5}}$ | $\boldsymbol{a}_{\boldsymbol{6}}$ | $\boldsymbol{a}_{\boldsymbol{7}}$ | $\boldsymbol{a}_{\boldsymbol{8}}$ | $\boldsymbol{a}_{\boldsymbol{9}}$ | $\hat{\boldsymbol{n}}$**^*^** | $\hat{\boldsymbol{p}}$**^#^** | ${\hat{\boldsymbol{k}}}_{\text{min}}$ | $\boldsymbol{n}\boldsymbol{-}\hat{\boldsymbol{n}}$ |
| **52** | 711 | 201 | 61 | 19 | 5 | 2 | 1 | 0 | - | 1000 | 0.711 | 8 | 0 |
| **53** | 723 | 180 | 63 | 26 | 4 | 1 | 1 | 1 | - | 999 | 0.724 | 8 | 1 |
| **54** | 703 | 209 | 62 | 21 | 4 | 1 | 0 | 0 | - | 1000 | 0.703 | 8 | 0 |
| **55** | 692 | 220 | 57 | 24 | 3 | 2 | 1 | 1 | - | 1000 | 0.692 | 8 | 0 |
| **56** | 687 | 218 | 67 | 17 | 5 | 5 | 0 | 0 | - | 999 | 0.688 | 8 | 1 |
| **57** | 697 | 211 | 69 | 18 | 3 | 2 | 0 | 0 | - | 1000 | 0.697 | 8 | 0 |
| **58** | 710 | 227 | 43 | 10 | 7 | 2 | 0 | 0 | - | 999 | 0.711 | 8 | 1 |
| **59** | 708 | 209 | 53 | 18 | 7 | 2 | 1 | 2 | - | 1000 | 0.708 | 8 | 0 |
| **…** | … | … | … | … | … | … | … | … | … | … | … | … | … |
| **66** | 726 | 189 | 60 | 21 | 3 | 1 | 0 | 0 | - | 1000 | 0.726 | 8 | 0 |
| **67** | 705 | 199 | 65 | 23 | 5 | 2 | 0 | 0 | - | 999 | 0.706 | 8 | 1 |
| **68** | 700 | 221 | 52 | 19 | 7 | 1 | 0 | 0 | - | 1000 | 0.700 | 8 | 0 |
| **…** | … | … | … | … | … | … | … | … | … | … | … | … | … |
| **70** | 716 | 200 | 59 | 16 | 5 | 4 | 0 | 0 | - | 1000 | 0.716 | 8 | 0 |
| **71** | 711 | 195 | 72 | 18 | 2 | 1 | 0 | 0 | - | 999 | 0.712 | 8 | 1 |
| **72** | 691 | 215 | 71 | 15 | 7 | 1 | 0 | 0 | - | 1000 | 0.691 | 8 | 0 |
| **…** | … | … | … | … | … | … | … | … | … | … | … | … | … |
| **98** | 710 | 211 | 53 | 18 | 6 | 1 | 0 | 0 | - | 999 | 0.711 | 8 | 1 |
| **99** | 709 | 212 | 55 | 16 | 4 | 2 | 2 | 0 | - | 1000 | 0.709 | 8 | 0 |
| **ID** | $\boldsymbol{a}_{\boldsymbol{1}}$ | $\boldsymbol{a}_{\boldsymbol{2}}$ | $\boldsymbol{a}_{\boldsymbol{3}}$ | $\boldsymbol{a}_{\boldsymbol{4}}$ | $\boldsymbol{a}_{\boldsymbol{5}}$ | $\boldsymbol{a}_{\boldsymbol{6}}$ | $\boldsymbol{a}_{\boldsymbol{7}}$ | $\boldsymbol{a}_{\boldsymbol{8}}$ | $\boldsymbol{a}_{\boldsymbol{9}}$ | $\hat{\boldsymbol{n}}$**^*^** | $\hat{\boldsymbol{p}}$**^#^** | ${\hat{\boldsymbol{k}}}_{\text{min}}$ | $\boldsymbol{n}\boldsymbol{-}\hat{\boldsymbol{n}}$ |
| **100** | 684 | 220 | 76 | 11 | 7 | 1 | 1 | 0 | 0 | 1000 | 0.684 | 9 | 0 |
| **Mean** | 700.44 | 207.74 | 63.74 | 19.04 | 6.10 | 1.92 | 0.63 | 0.27 | 0.154 | 999.89 | 0.70 | 8.10 | 0.11 |

**^*^**$\hat{\boldsymbol{n}}\boldsymbol{=}\boldsymbol{a}_{\boldsymbol{1}}\boldsymbol{+}\boldsymbol{a}_{\boldsymbol{2}}\boldsymbol{+\cdots}\boldsymbol{a}_{\boldsymbol{k}_{\text{mini}}}$**; ^#^**$\hat{\boldsymbol{p}}\boldsymbol{=}\boldsymbol{a}_{\boldsymbol{1}}\boldsymbol{/}\hat{\boldsymbol{n}}$

| **Table B1.6 Summary for** $\boldsymbol{p}_{\boldsymbol{i}}\boldsymbol{\sim N(0.7,0.0}3^{\boldsymbol{2}}\boldsymbol{)}$**, *n* =1000, 500, 100, 50** | | | | | |
| --- | --- | --- | --- | --- | --- |
|  | **Mean and 95% Confidence interval** | | | | |
| $\boldsymbol{n}$ | $\hat{\boldsymbol{n}}$ | $\hat{\boldsymbol{p}}$ | ${\hat{\boldsymbol{k}}}_{\text{min}}$ | $\boldsymbol{n}\boldsymbol{-}\hat{\boldsymbol{n}}$ | $\frac{\boldsymbol{n}\boldsymbol{-}\hat{\boldsymbol{n}}}{\boldsymbol{n}}$ |
| **1000** | 999.89±0.06 | 0.70±0.003 | 8.10±0.08 | 0.11±0.06 | 0.00011±0.00006 |
| **500** | 499.95±0.04 | 0.70±0.004 | 8.08±0.10 | 0.05±0.04 | 0.00010±0.00009 |
| **100** | 99.99±0.02 | 0.70±0.009 | 8.11±0.21 | 0.01±0.02 | 0.00010±0.00020 |
| **50** | 49.97±0.03 | 0.70±0.012 | 8.24±0.30 | 0.03±0.03 | 0.00060±0.00067 |

**Table B1.7 Summary for** $p_{i}\sim U(0.69,0.71)$**, *n* =1000**

| **ID** | $\boldsymbol{a}_{\boldsymbol{1}}$ | $\boldsymbol{a}_{\boldsymbol{2}}$ | $\boldsymbol{a}_{\boldsymbol{3}}$ | $\boldsymbol{a}_{\boldsymbol{4}}$ | $\boldsymbol{a}_{\boldsymbol{5}}$ | $\boldsymbol{a}_{\boldsymbol{6}}$ | $\boldsymbol{a}_{\boldsymbol{7}}$ | $\boldsymbol{a}_{\boldsymbol{8}}$ | $\boldsymbol{a}_{\boldsymbol{9}}$ | $\hat{\boldsymbol{n}}$**^*^** | $\hat{\boldsymbol{p}}$**^#^** | ${\hat{\boldsymbol{k}}}_{\text{min}}$ | $\boldsymbol{n}\boldsymbol{-}\hat{\boldsymbol{n}}$ |
| --- | --- | --- | --- | --- | --- | --- | --- | --- | --- | --- | --- | --- | --- |
| **1** | 701 | 218 | 63 | 9 | 8 | 0 | 1 | 0 | - | 1000 | 0.701 | 8 | 0 |
| **2** | 724 | 203 | 52 | 14 | 7 | 0 | 0 | 0 | - | 1000 | 0.724 | 8 | 0 |
| **3** | 676 | 226 | 68 | 21 | 6 | 1 | 1 | 1 | 0 | 1000 | 0.676 | 9 | 0 |
| **…** | … | … | … | … | … | … | … | … | … | … | … | … | … |
| **12** | 700 | 201 | 72 | 17 | 8 | 0 | 1 | 1 | - | 1000 | 0.700 | 8 | 0 |
| **13** | 700 | 207 | 66 | 21 | 5 | 0 | 0 | 0 | - | 999 | 0.701 | 8 | 1 |
| **14** | 697 | 225 | 56 | 14 | 5 | 3 | 0 | 0 | - | 1000 | 0.697 | 8 | 0 |
| **…** | … | … | … | … | … | … | … | … | … | … | … | … | … |
| **25** | 681 | 226 | 65 | 15 | 6 | 4 | 1 | 2 | 0 | 1000 | 0.681 | 9 | 0 |
| **26** | 702 | 208 | 64 | 19 | 4 | 1 | 1 | 0 | - | 999 | 0.703 | 8 | 1 |
| **27** | 719 | 195 | 57 | 21 | 4 | 2 | 1 | 0 | - | 999 | 0.720 | 8 | 1 |
| **28** | 721 | 201 | 52 | 14 | 10 | 2 | 0 | 0 | - | 1000 | 0.721 | 8 | 0 |
| **…** | … | … | … | … | … | … | … | … | … | … | … | … | … |
| **40** | 703 | 211 | 65 | 13 | 4 | 4 | 0 | 0 | - | 1000 | 0.703 | 8 | 0 |
| **41** | 713 | 194 | 62 | 19 | 7 | 2 | 0 | 2 | - | 999 | 0.714 | 8 | 1 |
| **42** | 696 | 203 | 68 | 28 | 1 | 1 | 2 | 0 | - | 999 | 0.697 | 8 | 1 |
| **43** | 691 | 218 | 69 | 16 | 5 | 1 | 0 | 0 | - | 1000 | 0.691 | 8 | 0 |
| **…** | … | … | … | … | … | … | … | … | … | … | … | … | … |
| **ID** | $\boldsymbol{a}_{\boldsymbol{1}}$ | $\boldsymbol{a}_{\boldsymbol{2}}$ | $\boldsymbol{a}_{\boldsymbol{3}}$ | $\boldsymbol{a}_{\boldsymbol{4}}$ | $\boldsymbol{a}_{\boldsymbol{5}}$ | $\boldsymbol{a}_{\boldsymbol{6}}$ | $\boldsymbol{a}_{\boldsymbol{7}}$ | $\boldsymbol{a}_{\boldsymbol{8}}$ | $\boldsymbol{a}_{\boldsymbol{9}}$ | $\hat{\boldsymbol{n}}$**^*^** | $\hat{\boldsymbol{p}}$**^#^** | ${\hat{\boldsymbol{k}}}_{\text{min}}$ | $\boldsymbol{n}\boldsymbol{-}\hat{\boldsymbol{n}}$ |
| **57** | 709 | 207 | 62 | 15 | 4 | 3 | 0 | 0 | -- | 1000 | 0.709 | 8 | 0 |
| **58** | 735 | 174 | 66 | 13 | 7 | 2 | 2 | - | - | 999 | 0.736 | 7 | 1 |
| **59** | 702 | 210 | 56 | 22 | 8 | 2 | 0 | 0 | - | 1000 | 0.702 | 8 | 0 |
| **…** | … | … | … | … | … | … | … | … | … | … | … | … | … |
| **62** | 720 | 194 | 59 | 16 | 8 | 2 | 1 | 0 | - | 1000 | 0.72 | 8 | 0 |
| **63** | 700 | 218 | 64 | 14 | 2 | 1 | 0 | 0 | - | 999 | 0.701 | 8 | 1 |
| **64** | 671 | 222 | 77 | 22 | 7 | 1 | 0 | 0 | 0 | 1000 | 0.671 | 9 | 0 |
| **…** | … | … | … | … | … | … | … | … | … | … | … | … | … |
| **69** | 711 | 192 | 72 | 21 | 2 | 2 | 0 | 0 | - | 1000 | 0.711 | 8 | 0 |
| **70** | 705 | 214 | 55 | 17 | 7 | 1 | 0 | 0 | - | 999 | 0.706 | 8 | 1 |
| **71** | 702 | 214 | 54 | 18 | 7 | 2 | 2 | 0 | - | 999 | 0.703 | 8 | 1 |
| **72** | 702 | 217 | 57 | 16 | 5 | 1 | 1 | 0 | - | 999 | 0.703 | 8 | 1 |
| **73** | 695 | 207 | 69 | 19 | 7 | 2 | 1 | 0 | - | 1000 | 0.695 | 8 | 0 |
| **…** | … | … | … | … | … | … | … | … | … | … | … | … | … |
| **99** | 713 | 203 | 63 | 12 | 6 | 2 | 1 | 0 | - | 1000 | 0.713 | 8 | 0 |
| **100** | 674 | 232 | 70 | 17 | 2 | 4 | 1 | 0 | 0 | 1000 | 0.674 | 9 | 0 |
| **Mean** | 699.25 | 210.96 | 62.76 | 18.86 | 5.83 | 1.6 | 0.48 | 0.15 | 0.07 | 999.90 | 0.699 | 8.13 | 0.10 |

**^*^**$\hat{\boldsymbol{n}}\boldsymbol{=}\boldsymbol{a}_{\boldsymbol{1}}\boldsymbol{+}\boldsymbol{a}_{\boldsymbol{2}}\boldsymbol{+\cdots}\boldsymbol{a}_{\boldsymbol{k}_{\text{mini}}}$**; ^#^**$\hat{\boldsymbol{p}}\boldsymbol{=}\boldsymbol{a}_{\boldsymbol{1}}\boldsymbol{/}\hat{\boldsymbol{n}}$

| **Table B1.8 Summary for**$\boldsymbol{p}_{\boldsymbol{i}}\boldsymbol{\sim U(0.6}9,\boldsymbol{0.7}1\boldsymbol{)}$**, *n* =1000, 500, 100, 50** | | | | | |
| --- | --- | --- | --- | --- | --- |
|  | **Mean and 95% Confidence interval** | | | | |
| $\boldsymbol{n}$ | $\hat{\boldsymbol{n}}$ | $\hat{\boldsymbol{p}}$ | ${\hat{\boldsymbol{k}}}_{\text{min}}$ | $\boldsymbol{n}\boldsymbol{-}\hat{\boldsymbol{n}}$ | $\frac{\boldsymbol{n}\boldsymbol{-}\hat{\boldsymbol{n}}}{\boldsymbol{n}}$ |
| **1000** | 999.90±0.06 | 0.70±0.003 | 8.13±0.07 | 0.10±0.06 | 0.00010±0.00006 |
| **500** | 499.99±0.02 | 0.70±0.005 | 8.11±0.11 | 0.01±0.02 | 0.00002±0.00004 |
| **100** | 99.98±0.03 | 0.70±0.009 | 8.19±0.23 | 0.02±0.03 | 0.00020±0.00028 |
| **50** | 50 | 0.70±0.013 | 8.22±0.30 | 0 | 0 |

**Table B1.9 Summary for** $p_{i}\sim U(0.67,0.73)$**, *n* =1000**

| **ID** | $\boldsymbol{a}_{\boldsymbol{1}}$ | $\boldsymbol{a}_{\boldsymbol{2}}$ | $\boldsymbol{a}_{\boldsymbol{3}}$ | $\boldsymbol{a}_{\boldsymbol{4}}$ | $\boldsymbol{a}_{\boldsymbol{5}}$ | $\boldsymbol{a}_{\boldsymbol{6}}$ | $\boldsymbol{a}_{\boldsymbol{7}}$ | $\boldsymbol{a}_{\boldsymbol{8}}$ | $\boldsymbol{a}_{\boldsymbol{9}}$ | $\hat{\boldsymbol{n}}$**^*^** | $\hat{\boldsymbol{p}}$**^#^** | ${\hat{\boldsymbol{k}}}_{\text{min}}$ | $\boldsymbol{n}\boldsymbol{-}\hat{\boldsymbol{n}}$ |
| --- | --- | --- | --- | --- | --- | --- | --- | --- | --- | --- | --- | --- | --- |
| **1** | 701 | 215 | 64 | 12 | 7 | 1 | 0 | 0 | - | 1000 | 0.70 | 8 | 0 |
| **2** | 731 | 198 | 50 | 14 | 6 | 1 | 0 | 0 | - | 1000 | 0.73 | 8 | 0 |
| **3** | 678 | 219 | 73 | 21 | 6 | 1 | 1 | 0 | 1 | 1000 | 0.68 | 9 | 0 |
| **…** | … | … | … | … | … | … | … | … | … | … | … | … | … |
| **19** | 683 | 217 | 79 | 15 | 5 | 0 | 1 | 0 | 0 | 1000 | 0.68 | 9 | 0 |
| **20** | 676 | 225 | 66 | 19 | 8 | 3 | 2 | 0 | 0 | 999 | 0.68 | 9 | 1 |
| **21** | 684 | 220 | 68 | 17 | 9 | 2 | 0 | 0 | 0 | 1000 | 0.68 | 9 | 0 |
| **…** | … | … | … | … | … | … | … | … | … | … | … | … | … |
| **30** | 717 | 195 | 55 | 22 | 6 | 4 | 1 | 0 | - | 1000 | 0.72 | 8 | 0 |
| **31** | 696 | 208 | 68 | 20 | 4 | 2 | 0 | 1 | - | 999 | 0.70 | 8 | 1 |
| **32** | 703 | 204 | 60 | 19 | 6 | 6 | 1 | 1 | - | 1000 | 0.70 | 8 | 0 |
| **33** | 685 | 206 | 77 | 22 | 9 | 1 | 0 | 0 | - | 1000 | 0.69 | 8 | 0 |
| **34** | 704 | 208 | 62 | 14 | 8 | 3 | 0 | 0 | - | 999 | 0.70 | 8 | 1 |
| **35** | 679 | 216 | 71 | 22 | 8 | 3 | 1 | 0 | 0 | 1000 | 0.68 | 9 | 0 |
| **…** | … | … | … | … | … | … | … | … | … | … | … | … | … |
| **43** | 690 | 220 | 67 | 12 | 8 | 2 | 0 | 1 | - | 1000 | 0.69 | 8 | 0 |
| **44** | 705 | 203 | 73 | 13 | 4 | 1 | 0 | 0 | - | 999 | 0.71 | 8 | 1 |
| **45** | 687 | 218 | 70 | 19 | 5 | 1 | 0 | 0 | - | 1000 | 0.69 | 8 | 0 |
| **ID** | $\boldsymbol{a}_{\boldsymbol{1}}$ | $\boldsymbol{a}_{\boldsymbol{2}}$ | $\boldsymbol{a}_{\boldsymbol{3}}$ | $\boldsymbol{a}_{\boldsymbol{4}}$ | $\boldsymbol{a}_{\boldsymbol{5}}$ | $\boldsymbol{a}_{\boldsymbol{6}}$ | $\boldsymbol{a}_{\boldsymbol{7}}$ | $\boldsymbol{a}_{\boldsymbol{8}}$ | $\boldsymbol{a}_{\boldsymbol{9}}$ | $\hat{\boldsymbol{n}}$**^*^** | $\hat{\boldsymbol{p}}$**^#^** | ${\hat{\boldsymbol{k}}}_{\text{min}}$ | $\boldsymbol{n}\boldsymbol{-}\hat{\boldsymbol{n}}$ |
| **…** | … | … | … | … | … | … | … | … | … | … | … | … | … |
| **67** | 719 | 202 | 56 | 17 | 5 | 0 | 0 | 1 | - | 1000 | 0.72 | 8 | 0 |
| **68** | 691 | 200 | 83 | 22 | 2 | 1 | 0 | 0 | - | 999 | 0.69 | 8 | 1 |
| **69** | 708 | 196 | 76 | 17 | 1 | 1 | 1 | 0 | - | 1000 | 0.71 | 8 | 0 |
| **…** | … | … | … | … | … | … | … | … | … | … | … | … | … |
| **98** | 682 | 226 | 72 | 14 | 3 | 2 | 0 | 1 | 0 | 1000 | 0.68 | 9 | 0 |
| **99** | 714 | 201 | 60 | 18 | 4 | 3 | 0 | 0 | - | 1000 | 0.71 | 8 | 0 |
| **100** | 669 | 233 | 71 | 17 | 7 | 2 | 0 | 1 | 0 | 1000 | 0.67 | 9 | 0 |
| **Mean** | 699.75 | 209.97 | 63.27 | 18.31 | 6.3 | 1.7 | 0.47 | 0.17 | 0.07 | 999.95 | 0.70 | 8.14 | 0.05 |

**^*^**$\hat{\boldsymbol{n}}\boldsymbol{=}\boldsymbol{a}_{\boldsymbol{1}}\boldsymbol{+}\boldsymbol{a}_{\boldsymbol{2}}\boldsymbol{+\cdots}\boldsymbol{a}_{\boldsymbol{k}_{\text{mini}}}$**; ^#^**$\hat{\boldsymbol{p}}\boldsymbol{=}\boldsymbol{a}_{\boldsymbol{1}}\boldsymbol{/}\hat{\boldsymbol{n}}$

| **Table B1.10 Summary for**$\boldsymbol{p}_{\boldsymbol{i}}\boldsymbol{\sim U(0.67,0.73)}$**, *n* =1000, 500, 100, 50** | | | | | |
| --- | --- | --- | --- | --- | --- |
|  | **Mean and 95% Confidence interval** | | | | |
| $\boldsymbol{n}$ | $\hat{\boldsymbol{n}}$ | $\hat{\boldsymbol{p}}$ | ${\hat{\boldsymbol{k}}}_{\text{min}}$ | $\boldsymbol{n}\boldsymbol{-}\hat{\boldsymbol{n}}$ | $\frac{\boldsymbol{n}\boldsymbol{-}\hat{\boldsymbol{n}}}{\boldsymbol{n}}$ |
| **1000** | 999.95±0.04 | 0.70±0.003 | 8.14±0.07 | 0.05±0.04 | 0.00005±0.00004 |
| **500** | 499.97±0.03 | 0.70±0.005 | 8.13±0.12 | 0.03±0.03 | 0.00006±0.00007 |
| **100** | 100 | 0.70±0.010 | 8.22±0.24 | 0 | 0 |
| **50** | 49.99±0.02 | 0.70±0.013 | 8.28±0.31 | 0.01±0.02 | 0.00020±0.00039 |

**Table B2.1 Summary for** $\boldsymbol{p}_{\boldsymbol{i}}\boldsymbol{=p=0.5}$**, *n* =1000, 500, 100, 50**

|  | **Mean and 95% Confidence interval** | | | | |
| --- | --- | --- | --- | --- | --- |
| $\boldsymbol{n}$ | $\hat{\boldsymbol{n}}$ | $\hat{\boldsymbol{p}}$ | ${\hat{\boldsymbol{k}}}_{\text{min}}$ | $\boldsymbol{n}\boldsymbol{-}\hat{\boldsymbol{n}}$ | $\frac{\boldsymbol{n}\boldsymbol{-}\hat{\boldsymbol{n}}}{\boldsymbol{n}}$ |
| **1000** | 999.90±0.06 | 0.50±0.004 | 13.73±0.17 | 0.10±0.06 | 0.00010±0.00006 |
| **500** | 500 | 0.50±0.004 | 13.95±0.17 | 0 | 0 |
| **100** | 99.99±0.02 | 0.51±0.010 | 13.8±0.45 | 0.01±0.02 | 0.00010±0.00020 |
| **50** | 49.70±0.41 | 0.52±0.013 | 13.27±0.63 | 0.30±0.41 | 0.00600±0.00829 |

**Table B2.2 Summary for** $\boldsymbol{p}_{\boldsymbol{i}}\boldsymbol{=p=0.6}$**, *n* =1000, 500, 100, 50**

|  | **Mean and 95% Confidence interval** | | | | |
| --- | --- | --- | --- | --- | --- |
| $\boldsymbol{n}$ | $\hat{\boldsymbol{n}}$ | $\hat{\boldsymbol{p}}$ | ${\hat{\boldsymbol{k}}}_{\text{min}}$ | $\boldsymbol{n}\boldsymbol{-}\hat{\boldsymbol{n}}$ | $\frac{\boldsymbol{n}\boldsymbol{-}\hat{\boldsymbol{n}}}{\boldsymbol{n}}$ |
| **1000** | 999.94±0.05 | 0.60±0.003 | 10.63±0.11 | 0.06±0.05 | 0.00006±0.00005 |
| **500** | 499.96±0.04 | 0.60±0.005 | 10.62±0.14 | 0.04±0.04 | 0.00008±0.00008 |
| **100** | 100 | 0.61±0.010 | 10.47±0.29 | 0 | 0 |
| **50** | 49.99±0.02 | 0.60±0.014 | 10.82±0.44 | 0.01±0.02 | 0.00020±0.00039 |

**Table B2.3 Summary for** $\boldsymbol{p}_{\boldsymbol{i}}\boldsymbol{=p=0.8}$**, *n* =1000, 500, 100, 50**

|  | **Mean and 95% Confidence interval** | | | | |
| --- | --- | --- | --- | --- | --- |
| $\boldsymbol{n}$ | $\hat{\boldsymbol{n}}$ | $\hat{\boldsymbol{p}}$ | ${\hat{\boldsymbol{k}}}_{\text{min}}$ | $\boldsymbol{n}\boldsymbol{-}\hat{\boldsymbol{n}}$ | $\frac{\boldsymbol{n}\boldsymbol{-}\hat{\boldsymbol{n}}}{\boldsymbol{n}}$ |
| **1000** | 999.91±0.06 | 0.80±0.003 | 6.10±0.06 | 0.09±0.06 | 0.00009±0.00006 |
| **500** | 499.97±0.03 | 0.80±0.003 | 6.24±0.09 | 0.03±0.03 | 0.00006±0.00007 |
| **100** | 99.97±0.03 | 0.81±0.008 | 6.14±0.16 | 0.03±0.03 | 0.00030±0.00034 |
| **50** | 49.97±0.04 | 0.81±0.011 | 6.17±0.23 | 0.03±0.04 | 0.0006±0.00087 |

**Table B2.4 Summary for** $\boldsymbol{p}_{\boldsymbol{i}}\boldsymbol{=p=0.9}$**, *n* =1000, 500, 100, 50**

|  | **Mean and 95% Confidence interval** | | | | |
| --- | --- | --- | --- | --- | --- |
| $\boldsymbol{n}$ | $\hat{\boldsymbol{n}}$ | $\hat{\boldsymbol{p}}$ | ${\hat{\boldsymbol{k}}}_{\text{min}}$ | $\boldsymbol{n}\boldsymbol{-}\hat{\boldsymbol{n}}$ | $\frac{\boldsymbol{n}\boldsymbol{-}\hat{\boldsymbol{n}}}{\boldsymbol{n}}$ |
| **1000** | 999.91±0.07 | 0.90±0.002 | 4.55±0.10 | 0.09±0.07 | 0.00009±0.00007 |
| **500** | 500 | 0.90±0.003 | 4.52±0.10 | 0 | 0 |
| **100** | 100 | 0.90±0.006 | 4.51±0.12 | 0 | 0 |
| **50** | 50 | 0.90±0.009 | 4.46±0.18 | 0 | 0 |

| **Table B3.1 Summary for** $\boldsymbol{p}_{\boldsymbol{i}}\boldsymbol{\sim N(0.5,0.0}\boldsymbol{1}^{\boldsymbol{2}}\boldsymbol{)}$**, *n* =1000, 500, 100, 50** | | | | | |
| --- | --- | --- | --- | --- | --- |
|  | **Mean and 95% Confidence interval** | | | | |
| $\boldsymbol{n}$ | $\hat{\boldsymbol{n}}$ | $\hat{\boldsymbol{p}}$ | ${\hat{\boldsymbol{k}}}_{\text{min}}$ | $\boldsymbol{n}\boldsymbol{-}\hat{\boldsymbol{n}}$ | $\frac{\boldsymbol{n}\boldsymbol{-}\hat{\boldsymbol{n}}}{\boldsymbol{n}}$ |
| **1000** | 999.97±0.03 | 0.50±0.003 | 13.90±0.14 | 0.03±0.03 | 0.00003±0.00003 |
| **500** | 499.94±0.05 | 0.50±0.004 | 13.81±0.19 | 0.06±0.05 | 0.00012±0.00009 |
| **100** | 99.98±0.03 | 0.49±0.009 | 14.28±0.39 | 0.02±0.03 | 0.00020±0.00028 |
| **50** | 49.20±0.61 | 0.50±0.014 | 13.42±0.83 | 0.80±0.61 | 0.01600±0.01218 |

| **Table B3.2 Summary for** $\boldsymbol{p}_{\boldsymbol{i}}\boldsymbol{\sim N(0.6,0.0}\boldsymbol{1}^{\boldsymbol{2}}\boldsymbol{)}$**, *n* =1000, 500, 100, 50** | | | | | |
| --- | --- | --- | --- | --- | --- |
|  | **Mean and 95% Confidence interval** | | | | |
| $\boldsymbol{n}$ | $\hat{\boldsymbol{n}}$ | $\hat{\boldsymbol{p}}$ | ${\hat{\boldsymbol{k}}}_{\text{min}}$ | $\boldsymbol{n}\boldsymbol{-}\hat{\boldsymbol{n}}$ | $\frac{\boldsymbol{n}\boldsymbol{-}\hat{\boldsymbol{n}}}{\boldsymbol{n}}$ |
| **1000** | 999.88±0.06 | 0.60±0.003 | 10.60±0.11 | 0.12±0.06 | 0.00012±0.00006 |
| **500** | 499.98±0.03 | 0.60±0.005 | 10.64±0.15 | 0.02±0.03 | 0.00004±0.00006 |
| **100** | 99.99±0.02 | 0.60±0.009 | 10.87±0.27 | 0.01±0.02 | 0.00010±0.00020 |
| **50** | 49.92±0.10 | 0.61±0.015 | 10.54±0.46 | 0.08±0.10 | 0.00160±0.00206 |

| **Table B3.3 Summary for** $\boldsymbol{p}_{\boldsymbol{i}}\boldsymbol{\sim N(0.8,0.0}\boldsymbol{1}^{\boldsymbol{2}}\boldsymbol{)}$**, *n* =1000, 500, 100, 50** | | | | | |
| --- | --- | --- | --- | --- | --- |
|  | **Mean and 95% Confidence interval** | | | | |
| $\boldsymbol{n}$ | $\hat{\boldsymbol{n}}$ | $\hat{\boldsymbol{p}}$ | ${\hat{\boldsymbol{k}}}_{\text{min}}$ | $\boldsymbol{n}\boldsymbol{-}\hat{\boldsymbol{n}}$ | $\frac{\boldsymbol{n}\boldsymbol{-}\hat{\boldsymbol{n}}}{\boldsymbol{n}}$ |
| **1000** | 999.94±0.05 | 0.80±0.002 | 6.09±0.06 | 0.06±0.05 | 0.00006±0.00005 |
| **500** | 499.98±0.03 | 0.80±0.003 | 6.23±0.09 | 0.02±0.03 | 0.00004±0.00006 |
| **100** | 100 | 0.80±0.008 | 6.36±0.16 | 0 | 0 |
| **50** | 50 | 0.80±0.011 | 6.23±0.21 | 0 | 0 |

| **Table B3.4 Summary for** $\boldsymbol{p}_{\boldsymbol{i}}\boldsymbol{\sim N(0.9,0.0}\boldsymbol{1}^{\boldsymbol{2}}\boldsymbol{)}$**, *n* =1000, 500, 100, 50** | | | | | |
| --- | --- | --- | --- | --- | --- |
|  | **Mean and 95% Confidence interval** | | | | |
| $\boldsymbol{n}$ | $\hat{\boldsymbol{n}}$ | $\hat{\boldsymbol{p}}$ | ${\hat{\boldsymbol{k}}}_{\text{min}}$ | $\boldsymbol{n}\boldsymbol{-}\hat{\boldsymbol{n}}$ | $\frac{\boldsymbol{n}\boldsymbol{-}\hat{\boldsymbol{n}}}{\boldsymbol{n}}$ |
| **1000** | 999.97±0.03 | 0.90±0.002 | 4.54±0.10 | 0.03±0.03 | 0.00003±0.00003 |
| **500** | 499.99±0.02 | 0.90±0.002 | 4.52±0.10 | 0.01±0.02 | 0.00002±0.00004 |
| **100** | 100 | 0.89±0.006 | 4.70±0.12 | 0 | 0 |
| **50** | 49.99±0.02 | 0.90±0.008 | 4.48±0.16 | 0.01±0.02 | 0.00020±0.00039 |

| **Table B4.1 Summary for** $\boldsymbol{p}_{\boldsymbol{i}}\boldsymbol{\sim N(0.5,0.0}\boldsymbol{2}^{\boldsymbol{2}}\boldsymbol{)}$**, *n* =1000, 500, 100, 50** | | | | | |
| --- | --- | --- | --- | --- | --- |
|  | **Mean and 95% Confidence interval** | | | | |
| $\boldsymbol{n}$ | $\hat{\boldsymbol{n}}$ | $\hat{\boldsymbol{p}}$ | ${\hat{\boldsymbol{k}}}_{\text{min}}$ | $\boldsymbol{n}\boldsymbol{-}\hat{\boldsymbol{n}}$ | $\frac{\boldsymbol{n}\boldsymbol{-}\hat{\boldsymbol{n}}}{\boldsymbol{n}}$ |
| **1000** | 999.89±0.06 | 0.50±0.004 | 13.73±0.16 | 0.11±0.06 | 0.00011±0.00006 |
| **500** | 499.96±0.04 | 0.50±0.004 | 13.73±0.18 | 0.04±0.04 | 0.00008±0.00008 |
| **100** | 99.99±0.02 | 0.50±0.009 | 13.97±0.41 | 0.01±0.02 | 0.00010±0.00020 |
| **50** | 49.71±0.37 | 0.50±0.013 | 14.18±0.69 | 0.29±0.37 | 0.00580±0.00746 |

| **Table B4.2 Summary for** $\boldsymbol{p}_{\boldsymbol{i}}\boldsymbol{\sim N(0.6,0.0}\boldsymbol{2}^{\boldsymbol{2}}\boldsymbol{)}$**, *n* =1000, 500, 100, 50** | | | | | |
| --- | --- | --- | --- | --- | --- |
|  | **Mean and 95% Confidence interval** | | | | |
| $\boldsymbol{n}$ | $\hat{\boldsymbol{n}}$ | $\hat{\boldsymbol{p}}$ | ${\hat{\boldsymbol{k}}}_{\text{min}}$ | $\boldsymbol{n}\boldsymbol{-}\hat{\boldsymbol{n}}$ | $\frac{\boldsymbol{n}\boldsymbol{-}\hat{\boldsymbol{n}}}{\boldsymbol{n}}$ |
| **1000** | 999.92±0.05 | 0.60±0.003 | 10.62±0.11 | 0.08±0.05 | 0.00008±0.00005 |
| **500** | 499.99±0.02 | 0.60±0.005 | 10.56±0.15 | 0.01±0.02 | 0.00002±0.00004 |
| **100** | 99.97±0.03 | 0.60±0.009 | 10.75±0.28 | 0.03±0.03 | 0.00030±0.00034 |
| **50** | 49.95±0.10 | 0.61±0.014 | 10.54±0.45 | 0.05±0.10 | 0.00100±0.00196 |

| **Table B4.3 Summary for** $\boldsymbol{p}_{\boldsymbol{i}}\boldsymbol{\sim N(0.7,0.0}\boldsymbol{2}^{\boldsymbol{2}}\boldsymbol{)}$**, *n* =1000, 500, 100, 50** | | | | | |
| --- | --- | --- | --- | --- | --- |
|  | **Mean and 95% Confidence interval** | | | | |
| $\boldsymbol{n}$ | $\hat{\boldsymbol{n}}$ | $\hat{\boldsymbol{p}}$ | ${\hat{\boldsymbol{k}}}_{\text{min}}$ | $\boldsymbol{n}\boldsymbol{-}\hat{\boldsymbol{n}}$ | $\frac{\boldsymbol{n}\boldsymbol{-}\hat{\boldsymbol{n}}}{\boldsymbol{n}}$ |
| **1000** | 999.90±0.06 | 0.70±0.003 | 8.14±0.08 | 0.10±0.06 | 0.00010±0.00006 |
| **500** | 499.95±0.04 | 0.70±0.004 | 8.08±0.11 | 0.05±0.04 | 0.00010±0.00009 |
| **100** | 99.98±0.03 | 0.70±0.009 | 8.14±0.21 | 0.02±0.03 | 0.00020±0.00028 |
| **50** | 50 | 0.72±0.014 | 7.82±0.31 | 0 | 0 |

| **Table B4.4 Summary for** $\boldsymbol{p}_{\boldsymbol{i}}\boldsymbol{\sim N(0.8,0.0}\boldsymbol{2}^{\boldsymbol{2}}\boldsymbol{)}$**, *n* =1000, 500, 100, 50** | | | | | |
| --- | --- | --- | --- | --- | --- |
|  | **Mean and 95% Confidence interval** | | | | |
| $\boldsymbol{n}$ | $\hat{\boldsymbol{n}}$ | $\hat{\boldsymbol{p}}$ | ${\hat{\boldsymbol{k}}}_{\text{min}}$ | $\boldsymbol{n}\boldsymbol{-}\hat{\boldsymbol{n}}$ | $\frac{\boldsymbol{n}\boldsymbol{-}\hat{\boldsymbol{n}}}{\boldsymbol{n}}$ |
| **1000** | 999.88±0.07 | 0.80±0.002 | 6.08±0.05 | 0.12±0.07 | 0.00012±0.00007 |
| **500** | 499.96±0.04 | 0.80±0.004 | 6.25±0.09 | 0.04±0.04 | 0.00008±0.00008 |
| **100** | 99.99±0.02 | 0.80±0.007 | 6.27±0.15 | 0.01±0.02 | 0.00010±0.00020 |
| **50** | 49.99±0.02 | 0.80±0.012 | 6.31±0.23 | 0.01±0.02 | 0.00020±0.00039 |

| **Table B4.5 Summary for** $\boldsymbol{p}_{\boldsymbol{i}}\boldsymbol{\sim N(0.9,0.0}\boldsymbol{2}^{\boldsymbol{2}}\boldsymbol{)}$**, *n* =1000, 500, 100, 50** | | | | | |
| --- | --- | --- | --- | --- | --- |
|  | **Mean and 95% Confidence interval** | | | | |
| $\boldsymbol{n}$ | $\hat{\boldsymbol{n}}$ | $\hat{\boldsymbol{p}}$ | ${\hat{\boldsymbol{k}}}_{\text{min}}$ | $\boldsymbol{n}\boldsymbol{-}\hat{\boldsymbol{n}}$ | $\frac{\boldsymbol{n}\boldsymbol{-}\hat{\boldsymbol{n}}}{\boldsymbol{n}}$ |
| **1000** | 999.89±0.07 | 0.90±0.002 | 4.50±0.10 | 0.11±0.07 | 0.00011±0.00007 |
| **500** | 499.98±0.03 | 0.90±0.003 | 4.51±0.10 | 0.02±0.03 | 0.00004±0.00006 |
| **100** | 99.99±0.02 | 0.90±0.006 | 4.60±0.12 | 0.01±0.02 | 0.00010±0.00020 |
| **50** | 50 | 0.90±0.007 | 4.45±0.15 | 0 | 0 |

| **Table B5.1 Summary for** $\boldsymbol{p}_{\boldsymbol{i}}\boldsymbol{\sim N(0.5,0.0}\boldsymbol{3}^{\boldsymbol{2}}\boldsymbol{)}$**, *n* =1000, 500, 100, 50** | | | | | |
| --- | --- | --- | --- | --- | --- |
|  | **Mean and 95% Confidence interval** | | | | |
| $\boldsymbol{n}$ | $\hat{\boldsymbol{n}}$ | $\hat{\boldsymbol{p}}$ | ${\hat{\boldsymbol{k}}}_{\text{min}}$ | $\boldsymbol{n}\boldsymbol{-}\hat{\boldsymbol{n}}$ | $\frac{\boldsymbol{n}\boldsymbol{-}\hat{\boldsymbol{n}}}{\boldsymbol{n}}$ |
| **1000** | 999.87±0.07 | 0.50±0.003 | 13.75±0.15 | 0.13±0.07 | 0.00013±0.00007 |
| **500** | 499.95±0.05 | 0.50±0.005 | 13.78±0.21 | 0.05±0.05 | 0.00010±0.00010 |
| **100** | 99.97±0.03 | 0.50±0.009 | 13.94±0.38 | 0.03±0.03 | 0.00030±0.00034 |
| **50** | 49.68±0.41 | 0.51±0.014 | 13.61±0.69 | 0.32±0.41 | 0.00640±0.00830 |

| **Table B5.2 Summary for** $\boldsymbol{p}_{\boldsymbol{i}}\boldsymbol{\sim N(0.6,0.0}\boldsymbol{3}^{\boldsymbol{2}}\boldsymbol{)}$**, *n* =1000, 500, 100, 50** | | | | | |
| --- | --- | --- | --- | --- | --- |
|  | **Mean and 95% Confidence interval** | | | | |
| $\boldsymbol{n}$ | $\hat{\boldsymbol{n}}$ | $\hat{\boldsymbol{p}}$ | ${\hat{\boldsymbol{k}}}_{\text{min}}$ | $\boldsymbol{n}\boldsymbol{-}\hat{\boldsymbol{n}}$ | $\frac{\boldsymbol{n}\boldsymbol{-}\hat{\boldsymbol{n}}}{\boldsymbol{n}}$ |
| **1000** | 999.90±0.06 | 0.60±0.003 | 10.63±0.11 | 0.10±0.06 | 0.00010±0.00006 |
| **500** | 499.96±0.04 | 0.60±0.005 | 10.58±0.15 | 0.04±0.04 | 0.00008±0.00008 |
| **100** | 99.98±0.03 | 0.60±0.008 | 10.76±0.26 | 0.02±0.03 | 0.00020±0.00028 |
| **50** | 49.92±0.10 | 0.60±0.013 | 10.56±0.44 | 0.08±0.10 | 0.00160±0.00206 |

| **Table B5.3 Summary for** $\boldsymbol{p}_{\boldsymbol{i}}\boldsymbol{\sim N(0.8,0.0}\boldsymbol{3}^{\boldsymbol{2}}\boldsymbol{)}$**, *n* =1000, 500, 100, 50** | | | | | |
| --- | --- | --- | --- | --- | --- |
|  | **Mean and 95% Confidence interval** | | | | |
| $\boldsymbol{n}$ | $\hat{\boldsymbol{n}}$ | $\hat{\boldsymbol{p}}$ | ${\hat{\boldsymbol{k}}}_{\text{min}}$ | $\boldsymbol{n}\boldsymbol{-}\hat{\boldsymbol{n}}$ | $\frac{\boldsymbol{n}\boldsymbol{-}\hat{\boldsymbol{n}}}{\boldsymbol{n}}$ |
| **1000** | 999.95±0.04 | 0.80±0.003 | 6.11±0.06 | 0.05±0.04 | 0.00005±0.00004 |
| **500** | 499.93±0.06 | 0.80±0.004 | 6.18±0.09 | 0.07±0.06 | 0.00014±0.00012 |
| **100** | 99.99±0.02 | 0.80±0.008 | 6.20±0.16 | 0.01±0.02 | 0.00010±0.00020 |
| **50** | 49.97±0.03 | 0.80±0.010 | 6.22±0.20 | 0.03±0.03 | 0.00060±0.00067 |

| **Table B5.4 Summary for** $\boldsymbol{p}_{\boldsymbol{i}}\boldsymbol{\sim N(0.9,0.0}\boldsymbol{3}^{\boldsymbol{2}}\boldsymbol{)}$**, *n* =1000, 500, 100, 50** | | | | | |
| --- | --- | --- | --- | --- | --- |
|  | **Mean and 95% Confidence interval** | | | | |
| $\boldsymbol{n}$ | $\hat{\boldsymbol{n}}$ | $\hat{\boldsymbol{p}}$ | ${\hat{\boldsymbol{k}}}_{\text{min}}$ | $\boldsymbol{n}\boldsymbol{-}\hat{\boldsymbol{n}}$ | $\frac{\boldsymbol{n}\boldsymbol{-}\hat{\boldsymbol{n}}}{\boldsymbol{n}}$ |
| **1000** | 999.84±0.08 | 0.90±0.002 | 4.50±0.10 | 0.16±0.08 | 0.00016±0.00008 |
| **500** | 499.90±0.07 | 0.90±0.002 | 4.46±0.10 | 0.10±0.07 | 0.00020±0.00013 |
| **100** | 100 | 0.89±0.006 | 4.59±0.12 | 0 | 0 |
| **50** | 50 | 0.90±0.007 | 4.48±0.16 | 0 | 0 |

| **Table B6.1 Summary for** $\boldsymbol{p}_{\boldsymbol{i}}\boldsymbol{\sim U(0.49,0.51)}$**, *n* =1000, 500, 100, 50** | | | | | |
| --- | --- | --- | --- | --- | --- |
|  | **Mean and 95% Confidence interval** | | | | |
| $\boldsymbol{n}$ | $\hat{\boldsymbol{n}}$ | $\hat{\boldsymbol{p}}$ | ${\hat{\boldsymbol{k}}}_{\text{min}}$ | $\boldsymbol{n}\boldsymbol{-}\hat{\boldsymbol{n}}$ | $\frac{\boldsymbol{n}\boldsymbol{-}\hat{\boldsymbol{n}}}{\boldsymbol{n}}$ |
| **1000** | 999.94±0.05 | 0.50±0.003 | 13.85±0.14 | 0.06±0.05 | 0.00006±0.00005 |
| **500** | 499.95±0.04 | 0.50±0.004 | 13.91±0.19 | 0.05±0.04 | 0.00010±0.00009 |
| **100** | 99.99±0.02 | 0.50±0.009 | 13.94±0.41 | 0.01±0.02 | 0.00010±0.00020 |
| **50** | 49.90±0.18 | 0.49±0.013 | 14.32±0.66 | 0.10±0.18 | 0.00200±0.00355 |

| **Table B6.2 Summary for** $\boldsymbol{p}_{\boldsymbol{i}}\boldsymbol{\sim U(0.59,0.61)}$**, *n* =1000, 500, 100, 50** | | | | | |
| --- | --- | --- | --- | --- | --- |
|  | **Mean and 95% Confidence interval** | | | | |
| $\boldsymbol{n}$ | $\hat{\boldsymbol{n}}$ | $\hat{\boldsymbol{p}}$ | ${\hat{\boldsymbol{k}}}_{\text{min}}$ | $\boldsymbol{n}\boldsymbol{-}\hat{\boldsymbol{n}}$ | $\frac{\boldsymbol{n}\boldsymbol{-}\hat{\boldsymbol{n}}}{\boldsymbol{n}}$ |
| **1000** | 999.87±0.07 | 0.60±0.004 | 10.50±0.11 | 0.13±0.07 | 0.00013±0.00007 |
| **500** | 499.95±0.04 | 0.60±0.005 | 10.58±0.14 | 0.05±0.04 | 0.00010±0.00009 |
| **100** | 99.98±0.03 | 0.60±0.009 | 10.65±0.28 | 0.02±0.03 | 0.00020±0.00028 |
| **50** | 49.93±0.12 | 0.61±0.014 | 10.45±0.47 | 0.07±0.12 | 0.00140±0.00238 |

| **Table B6.3 Summary for** $\boldsymbol{p}_{\boldsymbol{i}}\boldsymbol{\sim U(0.79,0.81)}$**, *n* =1000, 500, 100, 50** | | | | | |
| --- | --- | --- | --- | --- | --- |
|  | **Mean and 95% Confidence interval** | | | | |
| $\boldsymbol{n}$ | $\hat{\boldsymbol{n}}$ | $\hat{\boldsymbol{p}}$ | ${\hat{\boldsymbol{k}}}_{\text{min}}$ | $\boldsymbol{n}\boldsymbol{-}\hat{\boldsymbol{n}}$ | $\frac{\boldsymbol{n}\boldsymbol{-}\hat{\boldsymbol{n}}}{\boldsymbol{n}}$ |
| **1000** | 999.94±0.05 | 0.80±0.002 | 6.15±0.07 | 0.06±0.05 | 0.00006±0.00005 |
| **500** | 499.97±0.03 | 0.80±0.004 | 6.21±0.08 | 0.03±0.03 | 0.00006±0.00007 |
| **100** | 100 | 0.80±0.008 | 6.35±0.17 | 0 | 0 |
| **50** | 49.96±0.06 | 0.80±0.013 | 6.15±0.27 | 0.04±0.06 | 0.00080±0.00124 |

| **Table B6.4 Summary for** $\boldsymbol{p}_{\boldsymbol{i}}\boldsymbol{\sim U(0.89,0.91)}$**, *n* =1000, 500, 100, 50** | | | | | |
| --- | --- | --- | --- | --- | --- |
|  | **Mean and 95% Confidence interval** | | | | |
| $\boldsymbol{n}$ | $\hat{\boldsymbol{n}}$ | $\hat{\boldsymbol{p}}$ | ${\hat{\boldsymbol{k}}}_{\text{min}}$ | $\boldsymbol{n}\boldsymbol{-}\hat{\boldsymbol{n}}$ | $\frac{\boldsymbol{n}\boldsymbol{-}\hat{\boldsymbol{n}}}{\boldsymbol{n}}$ |
| **1000** | 999.97±0.03 | 0.90±0.002 | 4.49±0.10 | 0.03±0.03 | 0.00003±0.00003 |
| **500** | 499.95±0.04 | 0.90±0.003 | 4.48±0.10 | 0.05±0.04 | 0.00010±0.00009 |
| **100** | 99.98±0.03 | 0.90±0.006 | 4.56±0.13 | 0.02±0.03 | 0.00020±0.00028 |
| **50** | 49.97±0.03 | 0.90±0.009 | 4.51±0.19 | 0.03±0.03 | 0.00060±0.00067 |

| **Table B7.1 Summary for** $\boldsymbol{p}_{\boldsymbol{i}}\boldsymbol{\sim U(0.48,0.52)}$**, *n* =1000, 500, 100, 50** | | | | | |
| --- | --- | --- | --- | --- | --- |
|  | **Mean and 95% Confidence interval** | | | | |
| $\boldsymbol{n}$ | $\hat{\boldsymbol{n}}$ | $\hat{\boldsymbol{p}}$ | ${\hat{\boldsymbol{k}}}_{\text{min}}$ | $\boldsymbol{n}\boldsymbol{-}\hat{\boldsymbol{n}}$ | $\frac{\boldsymbol{n}\boldsymbol{-}\hat{\boldsymbol{n}}}{\boldsymbol{n}}$ |
| **1000** | 999.94±0.05 | 0.50±0.003 | 13.85±0.16 | 0.06±0.05 | 0.00006±0.00005 |
| **500** | 499.98±0.03 | 0.50±0.004 | 13.78±0.19 | 0.02±0.03 | 0.00004±0.00006 |
| **100** | 100 | 0.50±0.010 | 13.88±0.43 | 0 | 0 |
| **50** | 49.66±0.37 | 0.50±0.014 | 14.01±0.72 | 0.34±0.37 | 0.00680±0.00740 |

| **Table B7.2 Summary for** $\boldsymbol{p}_{\boldsymbol{i}}\boldsymbol{\sim U(0.58,0.62)}$**, *n* =1000, 500, 100, 50** | | | | | |
| --- | --- | --- | --- | --- | --- |
|  | **Mean and 95% Confidence interval** | | | | |
| $\boldsymbol{n}$ | $\hat{\boldsymbol{n}}$ | $\hat{\boldsymbol{p}}$ | ${\hat{\boldsymbol{k}}}_{\text{min}}$ | $\boldsymbol{n}\boldsymbol{-}\hat{\boldsymbol{n}}$ | $\frac{\boldsymbol{n}\boldsymbol{-}\hat{\boldsymbol{n}}}{\boldsymbol{n}}$ |
| **1000** | 999.92±0.05 | 0.60±0.003 | 10.56±0.12 | 0.08±0.05 | 0.00008±0.00005 |
| **500** | 499.96±0.04 | 0.60±0.005 | 10.57±0.15 | 0.04±0.04 | 0.00008±0.00008 |
| **100** | 99.99±0.02 | 0.60±0.010 | 10.60±0.30 | 0.01±0.02 | 0.00010±0.00020 |
| **50** | 50 | 0.61±0.015 | 10.62±0.47 | 0 | 0 |

| **Table B7.3 Summary for** $\boldsymbol{p}_{\boldsymbol{i}}\boldsymbol{\sim U(0.68,0.72)}$**, *n* =1000, 500, 100, 50** | | | | | |
| --- | --- | --- | --- | --- | --- |
|  | **Mean and 95% Confidence interval** | | | | |
| $\boldsymbol{n}$ | $\hat{\boldsymbol{n}}$ | $\hat{\boldsymbol{p}}$ | ${\hat{\boldsymbol{k}}}_{\text{min}}$ | $\boldsymbol{n}\boldsymbol{-}\hat{\boldsymbol{n}}$ | $\frac{\boldsymbol{n}\boldsymbol{-}\hat{\boldsymbol{n}}}{\boldsymbol{n}}$ |
| **1000** | 999.96±0.05 | 0.70±0.003 | 8.13±0.07 | 0.04±0.05 | 0.00004±0.00005 |
| **500** | 499.95±0.04 | 0.70±0.005 | 8.14±0.11 | 0.05±0.04 | 0.00010±0.00009 |
| **100** | 99.99±0.02 | 0.70±0.009 | 8.16±0.22 | 0.01±0.02 | 0.00010±0.00020 |
| **50** | 49.99±0.02 | 0.70±0.014 | 8.23±0.32 | 0.01±0.02 | 0.00020±0.00039 |

| **Table B7.4 Summary for** $\boldsymbol{p}_{\boldsymbol{i}}\boldsymbol{\sim U(0.78,0.82)}$**, *n* =1000, 500, 100, 50** | | | | | |
| --- | --- | --- | --- | --- | --- |
|  | **Mean and 95% Confidence interval** | | | | |
| $\boldsymbol{n}$ | $\hat{\boldsymbol{n}}$ | $\hat{\boldsymbol{p}}$ | ${\hat{\boldsymbol{k}}}_{\text{min}}$ | $\boldsymbol{n}\boldsymbol{-}\hat{\boldsymbol{n}}$ | $\frac{\boldsymbol{n}\boldsymbol{-}\hat{\boldsymbol{n}}}{\boldsymbol{n}}$ |
| **1000** | 999.97±0.03 | 0.80±0.002 | 6.15±0.07 | 0.03±0.03 | 0.00003±0.00003 |
| **500** | 499.98±0.03 | 0.80±0.004 | 6.22±0.08 | 0.02±0.03 | 0.00004±0.00006 |
| **100** | 100 | 0.80±0.008 | 6.32±0.15 | 0 | 0 |
| **50** | 49.98±0.03 | 0.80±0.012 | 6.21±0.23 | 0.02±0.03 | 0.00040±0.00055 |

| **Table B7.5 Summary for** $\boldsymbol{p}_{\boldsymbol{i}}\boldsymbol{\sim U(0.88,0.92)}$**, *n* =1000, 500, 100, 50** | | | | | |
| --- | --- | --- | --- | --- | --- |
|  | **Mean and 95% Confidence interval** | | | | |
| $\boldsymbol{n}$ | $\hat{\boldsymbol{n}}$ | $\hat{\boldsymbol{p}}$ | ${\hat{\boldsymbol{k}}}_{\text{min}}$ | $\boldsymbol{n}\boldsymbol{-}\hat{\boldsymbol{n}}$ | $\frac{\boldsymbol{n}\boldsymbol{-}\hat{\boldsymbol{n}}}{\boldsymbol{n}}$ |
| **1000** | 999.96±0.04 | 0.90±0.002 | 4.49±0.10 | 0.04±0.04 | 0.00004±0.00004 |
| **500** | 499.99±0.02 | 0.90±0.003 | 4.50±0.10 | 0.01±0.02 | 0.00002±0.00004 |
| **100** | 100 | 0.90±0.006 | 4.54±0.13 | 0 | 0 |
| **50** | 49.99±0.02 | 0.90±0.009 | 4.49±0.18 | 0.01±0.02 | 0.00020±0.00039 |

| **Table B8.1 Summary for** $\boldsymbol{p}_{\boldsymbol{i}}\boldsymbol{\sim U(0.47,0.53)}$**, *n* =1000, 500, 100, 50** | | | | | |
| --- | --- | --- | --- | --- | --- |
|  | **Mean and 95% Confidence interval** | | | | |
| $\boldsymbol{n}$ | $\hat{\boldsymbol{n}}$ | $\hat{\boldsymbol{p}}$ | ${\hat{\boldsymbol{k}}}_{\text{min}}$ | $\boldsymbol{n}\boldsymbol{-}\hat{\boldsymbol{n}}$ | $\frac{\boldsymbol{n}\boldsymbol{-}\hat{\boldsymbol{n}}}{\boldsymbol{n}}$ |
| **1000** | 999.87±0.09 | 0.50±0.003 | 13.83±0.13 | 0.13±0.09 | 0.00013±0.00009 |
| **500** | 499.98±0.03 | 0.50±0.004 | 13.89±0.19 | 0.02±0.03 | 0.00004±0.00006 |
| **100** | 99.97±0.03 | 0.51±0.010 | 13.72±0.40 | 0.03±0.03 | 0.00030±0.00034 |
| **50** | 49.99±0.02 | 0.50±0.014 | 14.31±0.60 | 0.01±0.02 | 0.00020±0.00039 |

| **Table B8.2 Summary for** $\boldsymbol{p}_{\boldsymbol{i}}\boldsymbol{\sim U(0.57,0.63)}$**, *n* =1000, 500, 100, 50** | | | | | |
| --- | --- | --- | --- | --- | --- |
|  | **Mean and 95% Confidence interval** | | | | |
| $\boldsymbol{n}$ | $\hat{\boldsymbol{n}}$ | $\hat{\boldsymbol{p}}$ | ${\hat{\boldsymbol{k}}}_{\text{min}}$ | $\boldsymbol{n}\boldsymbol{-}\hat{\boldsymbol{n}}$ | $\frac{\boldsymbol{n}\boldsymbol{-}\hat{\boldsymbol{n}}}{\boldsymbol{n}}$ |
| **1000** | 999.94±0.05 | 0.60±0.004 | 10.54±0.11 | 0.06±0.05 | 0.00006±0.00005 |
| **500** | 499.95±0.04 | 0.60±0.005 | 10.49±0.15 | 0.05±0.04 | 0.00010±0.00009 |
| **100** | 100 | 0.60±0.009 | 10.65±0.30 | 0 | 0 |
| **50** | 49.92±0.12 | 0.61±0.014 | 10.4±0.47 | 0.08±0.12 | 0.00160±0.00241 |

| **Table B8.3 Summary for**$\boldsymbol{p}_{\boldsymbol{i}}\boldsymbol{\sim U(0.77,0.83)}$**, *n* =1000, 500, 100, 50** | | | | | |
| --- | --- | --- | --- | --- | --- |
|  | **Mean and 95% Confidence interval** | | | | |
| $\boldsymbol{n}$ | $\hat{\boldsymbol{n}}$ | $\hat{\boldsymbol{p}}$ | ${\hat{\boldsymbol{k}}}_{\text{min}}$ | $\boldsymbol{n}\boldsymbol{-}\hat{\boldsymbol{n}}$ | $\frac{\boldsymbol{n}\boldsymbol{-}\hat{\boldsymbol{n}}}{\boldsymbol{n}}$ |
| **1000** | 999.95±0.04 | 0.80±0.002 | 6.11±0.06 | 0.05±0.04 | 0.00005±0.00004 |
| **500** | 499.97±0.03 | 0.80±0.004 | 6.22±0.09 | 0.03±0.03 | 0.00006±0.00007 |
| **100** | 99.98±0.03 | 0.80±0.008 | 6.33±0.16 | 0.02±0.03 | 0.00020±0.00028 |
| **50** | 49.98±0.03 | 0.80±0.012 | 6.10±0.23 | 0.02±0.03 | 0.00040±0.00055 |

| **Table B8.4 Summary for** $\boldsymbol{p}_{\boldsymbol{i}}\boldsymbol{\sim U(0.87,0.93)}$**, *n* =1000, 500, 100, 50** | | | | | |
| --- | --- | --- | --- | --- | --- |
|  | **Mean and 95% Confidence interval** | | | | |
| $\boldsymbol{n}$ | $\hat{\boldsymbol{n}}$ | $\hat{\boldsymbol{p}}$ | ${\hat{\boldsymbol{k}}}_{\text{min}}$ | $\boldsymbol{n}\boldsymbol{-}\hat{\boldsymbol{n}}$ | $\frac{\boldsymbol{n}\boldsymbol{-}\hat{\boldsymbol{n}}}{\boldsymbol{n}}$ |
| **1000** | 999.92±0.05 | 0.90±0.002 | 4.51±0.10 | 0.08±0.05 | 0.00008±0.00005 |
| **500** | 499.97±0.03 | 0.90±0.003 | 4.45±0.10 | 0.03±0.03 | 0.00006±0.00007 |

| **100** | 100 | 0.90±0.006 | 4.53±0.12 | 0 | 0 |
| --- | --- | --- | --- | --- | --- |
| **50** | 49.97±0.03 | 0.90±0.009 | 4.46±0.18 | 0.03±0.03 | 0.00060±0.00067 |
